# Improved, Odorless Access to Benzo[1,2-d;4,5-d′]- bis[1,3]dithioles and *Tert*-butyl Arylsulfides via C-S Cross Coupling

**DOI:** 10.3390/molecules25163666

**Published:** 2020-08-12

**Authors:** Kevin Kopp, Olav Schiemann, Nico Fleck

**Affiliations:** Institute of Physical and Theoretical Chemistry, Rheinische Friedrich-Wilhelms-University Bonn, Wegelerstr. 12, 53115 Bonn, Germany; kopp@pc.uni-bonn.de (K.K.); schiemann@pc.uni-bonn.de (O.S.)

**Keywords:** palladium catalysis, trityl radicals, thioketal, thioether, isothiouronium bromide

## Abstract

Benzo[1,2-d;4,5-d′]bis[1,3]dithioles are important building blocks within a range of functional materials such as fluorescent dyes, conjugated polymers, and stable trityl radicals. Access to these is usually gained via *tert*-butyl aryl sulfides, the synthesis of which requires the use of highly malodorous *tert*-butyl thiol and relies on S_N_Ar-chemistry requiring harsh reaction conditions, while giving low yields. In the present work, S-*tert*-butyl isothiouronium bromide is successfully applied as an odorless surrogate for *tert*-butyl thiol. The C-S bond formation is carried out under palladium catalysis with the thiolate formed in situ resulting in high yields of *tert*-butyl aryl sulfides. The subsequent formation of benzo[1,2-d;4,5-d′]bis[1,3]dithioles is here achieved with scandium(III)triflate, a less harmful reagent than the usually used Lewis acids, e.g., boron trifluoride or tetrafluoroboric acid. This enables a convenient and environmentally more compliant access to high yields of benzo[1,2-d;4,5-d′]bis[1,3]dithioles.

## 1. Introduction

**Figure 1:**
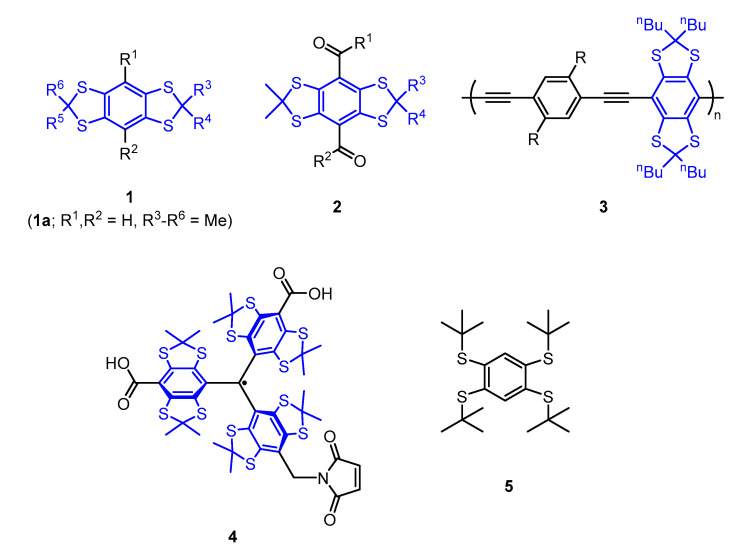
Lewis structures of benzo[1,2 d;4,5 d′]bis[1,3]dithioles **1**, “S4-DBD” dyes **2**, thioketal containing conjugated polymers **3**, spin label **4**^●^ and 1,2,4,5-tetrakis(tert-butylthio)benzene **5**. For the sake of clarity, the benzo[1,2-d;4,5-d′]bis[1,3]dithiole motif is highlighted in blue.

Benzo[1,2-d;4,5-d′]bis[1,3]dithioles **1** (Figure 1) have emerged as important building blocks for a range of functional materials. For example benzo[1,2-d:4,5-d′]bis[1,3]dithiole-based fluorescent dyes (“S4 DBD dyes” **2**) exhibit large stokes shifts making them promising candidates for super-resolution microscopy such as stimulated emission depletion (STED) microscopy [1,2]. Moreover, conjugated polymers **3** consisting of such building blocks show fluorescence response upon oxidation rendering these materials interesting for oxidant sensing [3]. Another widespread application lies in the synthesis of triarylmethyl radicals [4] (“TAM-radicals” **4**), a class of highly stable radicals employed in site-directed spin labeling of biomolecules for electron paramagnetic resonance (EPR)-based structure determination in vitro [5,6,7], at room temperature [5,6] and within cells [8,9,10]. Furthermore, such triaryl methyl (TAM)-radicals are used as sensors for local oxygen concentrations within EPR-imaging [11,12], as pH-probes [13], viscosimetry [14], and as polarizing agents for dynamic nuclear polarization (DNP) [15,16]. Additionally, TAM radicals incorporating benzo[1,2-d;4,5-d′]bis[1,3]dithiol moieties were recently taken into account for the design of magnetic materials and information transfer [17,18]. The synthesis of all of the thioketals shown in Figure 1 regularly involves 1,2,4,5-tetrakis(*tert*-butylthio)benzene **5** as the starting material [4], since the 1,2,4,5-tetrathiobenzene required for ketal formation easily undergoes oxidative degradation rendering its isolation cumbersome [19]. Precursor **5** is usually synthesized from 1,2,4,5-tetrachlorobenzene [4,20] via S_N_Ar-type reactions, which not only require harsh conditions, but also the use of excessive *tert*-butyl thiolate, generated from the free thiol and sodium [4] or sodium hydride [20] in situ. *Tert*-butylthiol however, is a quite volatile (Bp. 64 °C) and highly malodorous compound with an extremely low odor threshold of 0.33 ppb [21]. Considering the accompanied formation of H_2_ entraining free thiol into the gas-phase and the elevated temperatures of the reaction, the problematic nature of this synthetic step becomes apparent. In order to avoid harassment of the environment during this reaction, several strategies have been proposed in the literature, such as washing the gas-stream with KMnO_4_ [20] or thermal inactivation of the fume hood exhaust [22]. While the gas inlet can get clogged resulting in dangerous overpressures within the former approach, the latter one appears costly and requires a redesign of the fume hood exhaust system. This makes the synthesis of benzo[1,2-d;4,5-d′]bis[1,3]dithioles **1** rather unfit for e.g.,, academic laboratories. Additionally, precursor **5** is not commercially available in reasonable quantities, making a more convenient approach to aryl-*tert*-butyl thioether such as **5** highly desirable.

Considering the accomplishments in catalytic carbon-heteroatom-bond formation in form of Pd-catalyzed cross-coupling reactions [23], a similar approach would be desirable for the task here. There is a plethora of examples for catalytic C-S-bond formations [24,25,26,27,28,29], which hold some advantages as e.g., milder reaction conditions and better conversions as compared to classical S_N_Ar-type reactions. However, such an approach should avoid the use of free thiols altogether. Although C-S cross-coupling reactions using appropriate thiol surrogates are known, these usually need to be synthesized from the corresponding thiols, such as alkyl thioacetates [30]. Another possibility has been described by Wang et al. [31] in form of a one-pot synthesis, in which the necessary thiolate nucleophile was generated in situ using an alkyl bromide and thiourea as the sulfur source. Nonetheless, the yields for tertiary substrates stalled due to a competing elimination pathway.

In the present study, the olfactory hazard and lack of conversion was circumvented by synthesis of S-*tert*-butyl isothiouronium bromide from thiourea and *tert*-butyl bromide and its use as thiolate source in a subsequent cross-coupling reaction. Since the isothiouronium salt is completely odorless and the thiolate appears only in situ, the described process enables odorless access to benzo[1,2-d;4,5-d′]bis[1,3]dithioles and this with improved yields. It should be noted, that S-*tert*-butyl isothiouronium bromide has already been applied as a thiolate source for the functionalization of electron-poor heterocycles via nucleophilic substitution reactions [32,33]. Furthermore, a smoother conversion of **5** to the thioketal 2,2,6,6-tetramethyl- benzo[1,2-d;4,5-d′]bis[1,3]dithiole **1a** by use of catalytic amounts of Sc(OTf)_3_ was possible, which in comparison to previous protocols avoids the use of large amounts of hazardous Lewis acids like HBF_4_ and BF_3_.

## 2. Results and Discussion

Although it is well-known that S-alkyl isothiouronium salts form alkylthiols upon treatment with aqueous hydroxide [34], their behavior towards bases in nonaqueous media has been studied only rarely. An early report of Luzzio et al. [35] described the formation of thiolates from S-alkyl isothiouronium halides treated with NaH or NaOEt in refluxing THF or EtOH, respectively. Based on this, the capability of S-*tert*-butyl isothiouronium halides to form the corresponding thiolate exploitable for in-situ C-S cross coupling was assumed.

**Scheme 1:**
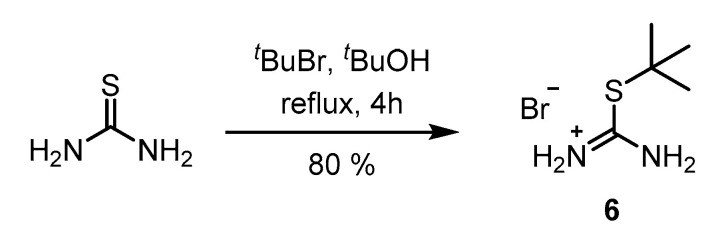
Synthesis of S-*tert*-butyl isothiouronium bromide.

Though S-*tert*-butyl isothiouronium halides were occasionally described in older literature [36], the procedures applied for their synthesis utilized gaseous hydrogen halides rendering them inconvenient and dangerous. In contrast, S-*n*-alkyl isothiouronium halides are easily prepared by reaction of the corresponding alkyl halide with thiourea in refluxing ethanol [34,37], but the competing elimination dominates the reactivity of tertiary alkyl halides inhibiting the formation of the desired S-*tert*-butyl isothiouronium salt. Accordingly, the reaction of thiourea with *tert*-butyl bromide in refluxing ethanol yielded pure S-ethyl isothiouronium bromide (Figure S3). This issue was circumvented by replacing EtOH for *t*-BuOH, so that the only electrophile in the reaction mixture was the *tert*-butyl group. Consequently, pure S-*tert*-butyl isothiouronium bromide **6** was obtained in a yield of 80% after recrystallization on a 10 g scale as shown in Scheme 1.

**Scheme 2:**
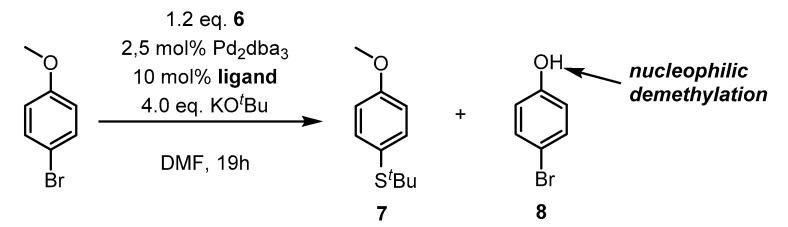
General reaction Scheme for the screening of optimal conversion of 4-bromoanisole.

With convenient access to the S-tert-butylisothiouronium salt **6** at hand, the performance of Pd-catalyzed C-S cross coupling of aryl bromides with *tert*-butyl thiolate generated in situ from **6** was examined. Aryl bromides were chosen as appropriate substrates, since cross-coupling reactions generally proceed worse with aryl chlorides and the aryl iodides are more expensive and commercially harder to come by. Inspired by an early publication by Migita et al. [25] an initial catalyst loading of 5 mol% of Pd catalyst with 10 mol% of phosphine ligand was chosen. We used the very common Pd_2_(dba)_3_ (2.5 mol%) as a Pd(0)-source, as dibenzylideneacetone is easily displaced by other ligands simplifying adjustments to the reaction conditions with regard to different phosphine ligands. Additionally, potassium *tert*-butoxide was used as a strong base for activation of the thiolate. Seeking for optimal conditions, 4-bromoanisole was subjected to C-S cross coupling tests using various conditions as shown in Scheme 2. 4-Bromoanisole was chosen, since no S_N_Ar background-reactivity was expected and the analysis of the reaction mixtures is easily possible via ^1^H-NMR. In order to gain sufficient reactivity, an initial temperature/ligand screening was performed giving the results shown in Table 1.

As evident from entries 1–3 (Table 1), low to no conversion of the substrate occurs at 50 °C. For cases where cross-coupling reactivity towards **7** was low, a competitive nucleophilic *O*-demethylation yielding **8** was observed. However, increasing the temperature to 80 °C gives quantitative conversion to the desired product **7** using Ph_3_P as a ligand (entry 4, Table 1). All other ligands show considerably less conversion at this temperature, decreasing in the order Ph_3_P > ^n^Bu_3_P > dppf >> Xantphos > XPhos/SPhos/BettPhos. Considering the steric demand of these ligands, the nucleophilic displacement of bromide by the thiolate anion in the catalytic cycle seems to be challenging. As for low reaction temperatures, the competitive O-demethylation (entries 5–10, Table 1) also for rather inactive catalysts [38]. As expected, no S_N_Ar background reaction was observed (Table 1, entries 11–13) and Pd_2_dba_3_ did not show any catalytic activity without phosphine ligands (Table 1, entry 11). Having established the combination Pd_2_dba_3_/Ph_3_P as a suitable catalyst system, the choice of base and solvent were assessed (Table 2).

While the transformation of 4-bromoanisole to **7** smoothly proceeds in DMF, the yield was diminished to 61% upon using *n*-butanol (Table 2, entry 1). As *n*-butanol is already a much less appropriate solvent for the formation of **7**, DMF was kept as the solvent and further screening of other bases was carried out in the latter. The use of milder bases such as Cs_2_CO_3_, K_2_CO_3_, or K_3_PO_4_ resulted in lower yields (Table 2, entries 2–6). Also, using reduced amounts of KO*^t^*Bu (Table 2, entry 7) dramatically reduces the yield. Interestingly, the yields were also significantly reduced by addition of 10 mol% 18-crown-6 to the reaction mixtures (Table 2, entries 4, 6).

**Scheme 3:**
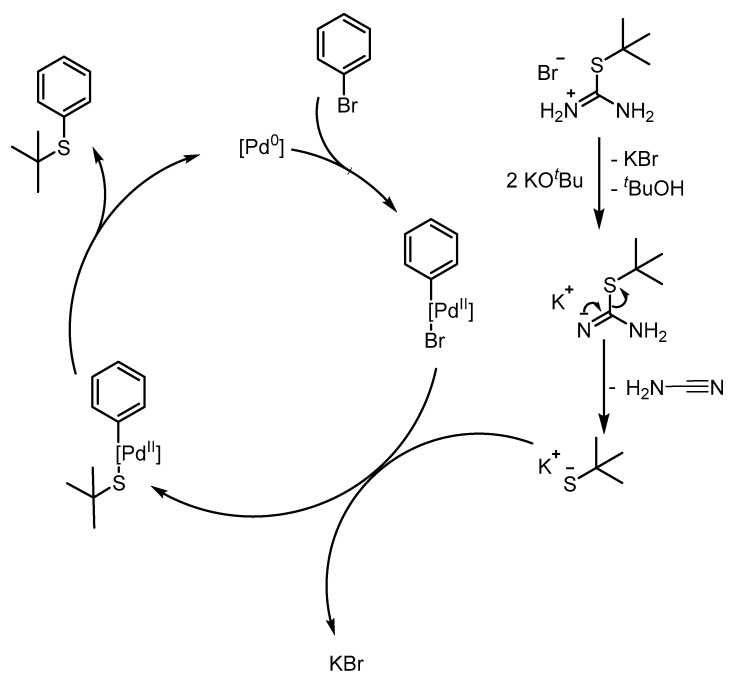
Assumed mechanism for the formation of *tert*-butyl thioethers from isothiouronium salts.

Thus, the use of KO*^t^*Bu, DMF and simple Ph_3_P enabled quantitative conversion of 4-bromoanisole to thioether **7**, while the reactivity with other ligands was significantly lower. At higher temperature, the cross-coupling reaction dominates and runs likely through a typical Pd^0^/Pd^II^ catalytic cycle (Scheme 3).

Considering the mechanism proposed in Scheme 3, thiolate formation is assumed to already occur at low temperature. This assumption is supported by the *O*-demethylation already occurring at 50 °C (Table 1, entries 1–3), which requires the presence of the thiolate anion. However, the nucleophilic displacement of bromide with the sterically demanding *tert*-butyl thiolate appears challenging, compared to the oxidative addition into the C_sp2_-Br bond, especially since the displacement is known to run through an associative pathway at the Pd^II^-center [39]. Thus, the substitution of bromide by the thiolate is suspected to be the rate-determining step in the catalytic cycle. This is further encouraged by the observation that the reaction readily proceeds in DMF, whereas the yield is heavily diminished in *n*-butanol. As the solubility of KBr in DMF is low [40], precipitation of the latter facilitates the nucleophilic displacement. The addition of a crown ether, however, increases both the solubility of KBr and the nucleophilicity of bromide, hampering this step and thereby the entire reaction.

With regard to the monodentate ligands no reactivity was observed when using biarylphosphines (XPhos, SPhos, BrettPhos; Table 1, entries 2, 7–9), despite their successful application for C-S cross coupling reactions in general [24]. The order of reactivity for the formation of **7** decreases in the order Ph_3_P > ^n^Bu_3_P > XPhos/BrettPhos/SPhos, while the respective cone angles increase in the reverse order Ph_3_P (131.4°) < ^n^Bu_3_P (152.2°) < SPhos (201.9°) < XPhos (209.8°) [41]. This observation can be rationalized through the associative displacement of bromide for thiolate being the rate-determining step. The steric congestion imposed by ligands with a large cone angle restrains the attack of the sterically demanding *tert*-butyl thiolate even further. In contrast and although performing considerably worse than Ph_3_P and ^n^Bu_3_P, the bidentate ligands dppf and XantPhos gave conversion to the desired product, with dppf performing about a factor 18 better than XantPhos. Though the exact coordination mode within the catalytic cycle is not known, the lower bite angle [42] (99°) of dppf is assumed to impose less steric congestion compared to XantPhos (108°), thereby facilitating the nucleophilic displacement step. Interestingly, XantPhos has been reported to catalyze C-S cross-coupling [26], however here only a very low reactivity was observed (Table 1, entries 3,6). Most of the recent studies on C-S cross-coupling with thiol surrogates focused on primary alkyl thiols, thus neglecting the influence of sterically demanding nucleophiles. Interestingly, the O-demethylation dominated especially with ligands, that render the nucleophilic displacement on the Pd^II^-center disfavored for steric reasons (vide infra). As the latter one is believed to be rate-determining, the cross-coupling is slowed down in these cases, so that educt reacts predominantly through the *O*-demethylation pathway.

Considering the aforementioned transformation of 4-bromoanisole so **7**, Park et al. [30] used a Pd(dba)_2_/dppf catalyst system and obtained a yield of 87% using S-*tert*-butylthioacetate as sulfur source, however with an increased temperature of 110 °C. Exploiting the in situ formation of thiolates from *tert*-butyl bromide and thiourea, Wang et al. [31] obtained S-*tert*-butylthiobenzene (yield 76%) and 4-nitro-S-*tert*-butylthiobenzene (yield 77%) from the respective aryl bromides using a Pd_2_dba_3_/XPhos catalyst system and yields were significantly higher for primary alkyl substrates.

To probe for a more general substrate applicability, 1,4-diiodobenzene, 4-bromonitrobenze, 4-chloroanisole, and 4-fluoroanisol were subjected to the desired transformation as shown in Table 3. For 1,4-diiodobenzene, quantitative conversion to the desired 1,4-bis(*tert*-butylthio)benzene was observed (conditions Table 1, entry 4) even at lower reaction temperature (50 °C). Though the nucleophilic displacement step (Scheme 3) is assumed to be rate determining for aryl bromides, the easier oxidative addition into the C-I bond seems to facilitate the desired transformation here. With 4-nitrobromobenzene, full conversion to the *tert-*butyl thioether was observed already at 25 °C (Table 3, entry 3). However, the same result was obtained without Pd-catalyst (Table 3, entry 4), which suggests that reaction proceeds in this case via an S_N_Ar-mechanism. This raises the question, whether the aforementioned result of Wang et al. relied on a true palladium catalyzed C-S cross coupling reaction or also on an S_N_Ar-reaction. In contrast, 4-chloroanisole (Table 3, entries 5,6) and 4-fluoroanisole (Table 3, entries 7,8) do yield no conversion to **7** regardless of type of catalyst. Instead O-demethylation occurs (Figures S36 and S37). Since Ph_3_P-ligated palladium species lack the capability of activating C_ar_-Cl and C_ar_-F bonds [29,39], both educts cannot enter the catalytic cycle presented in Scheme 3. In addition, 4-fluoro- and 4-chloroanisole do also not show any S_N_Ar-chemistry under the conditions used. Such a reaction maybe achieved with thiolate nucleophiles, e.g., by coordination to an electron withdrawing metal fragment [43], substitution by EWGs [44], or elevated temperatures [45].

**Scheme 4:**
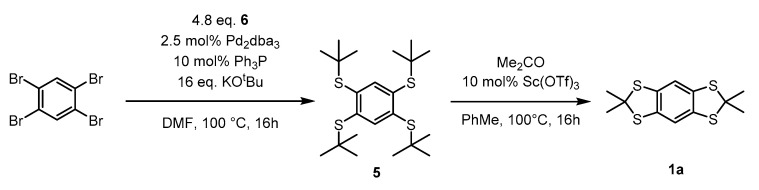
Synthesis of 2,2,6,6-Tetramethylbenzo[1,2-d:4,5-d′]bis[1,3]dithiole **1a**.

With proper reaction conditions at hand, 1,2,4,5-tetrabromobenzene was used as a substrate to obtain the desired 1,2,4,5-tetrakis(*tert*-butylthio)benzene **5** as shown in Scheme 4.

Here, the applicability of this transformation was further increased by reducing the catalyst load to 1.25 mol% Pd per coupling site and the desired product **5** was obtained in a yield of 88% on a 10 g scale after simple washing with methanol. This exceeds the literature yields varying between 48% [20] and 71% [46] significantly, while using the same isolation method. It should however be noted, that the transformation of 1,2,4,5-tetrabromobenzene to 5 proceeds with quantitative conversion (see Figure S6), proofed by quantitative ^1^H-NMR with mesitylene as internal standard.

The subsequent transformation to the thioketals is regularly carried out using an excess of BF_3_ or HBF_4_ [20,46], both reagents appear toxic and dangerous in handling. By contrast, thioketal **1a** could also be obtained in a yield of 83% on a 1 g scale by using catalytic amounts of Sc(OTf)_3_. Though yields of 96% were obtained with HBF_4_, and 78% with BF_3_ respectively [10], scandium(III)triflate appears undeniably as the safer and environmentally more benign reagent in this case.

## 3. Experimental

### 3.1. General

Commercially available chemicals were used without further purification, dry solvents were purchased in sealed containers over molecular sieves. All reactions involving air and water sensitive substrates were carried out under argon atmosphere using standard Schlenk techniques. Solvents were degassed by simple purging of the respective dry solvents with argon gas for 30 minutes. Observation of the reactions via thin layer chromatography was performed on aluminum silica plates with F254-fluorescence indicator obtained from Merck, the spots were visualized using 254 nm UV-light. Column chromatography was conducted using silica gel (60 Å pore size, 40–63 µm particle size) purchased from Merck. Solvents were removed under reduced pressure by a rotary evaporator.

### 3.2. Synthetic Procedures

S-*tert*-butyl isothiouronium bromide **6:** Thiourea (4.08 g, 53.6 mmol) was dissolved in *tert*-butanol (35 mL) and heated to reflux. Then *tert*-butyl bromide (9.76 g, 71.2 mmol, 1.32 eq.) was added and the mixture was stirred for 4 h under reflux. Completion of the reaction was determined by the absence of thiourea monitored by TLC (acetone/cyclohexane 10:1). The reaction mixture was then poured into cyclohexane whereupon a white solid precipitated, which was subsequently filtered off, washed with cold acetone and dried in high vacuum to afford the title compound as a white crystalline solid (9.16 g, 43.0 mmol, 80.2%). ^1^H-NMR (400 MHz, 298 K, DMSO-*d*_6_, δ in ppm): 1.49 (s, 9H), 9.15 (s, 4H). ^13^C-NMR (100 MHz, 298 K, DMSO-*d*_6_, δ in ppm): 30.9, 51.0, 166.0. HRMS (ESI+, *m*/*z*, [M − Br]^+^): calc. for C_5_H_13_N_2_S, 133.0794; found 133.0794.

4-Methoxy*-tert-*butylthiobenzene **7:** 4-Bromoanisole (1.00 g, 5.34 mmol), *tert*-butyl isothiouronium bromide (1.37 g, 6.43 mmol, 1.20 eq.), tris(dibenzylideneacetone)dipalladium(0) (0.12 g, 0.13 mmol, 2.5 mol%), triphenylphosphine (0.142 g, 0.54 mmol, 10 mol%) and potassium *tert*-butoxide (2.40 mg, 21.4 mmol, 4.00 eq.) were dissolved in dry and degassed DMF (40 mL) under argon atmosphere. The mixture was stirred for 19 h at 80 °C. Afterwards, the reaction was quenched with water (100 mL) and extracted with DCM (3 × 30 mL). The unified organic phases were repeatedly washed with 2M-HCl-solution (3 × 50 mL) and dried over MgSO_4_. The solvents were removed under reduced pressure and the crude product was subjected to column chromatography on silica eluting with cyclohexane/ethyl acetate 100:1 (*v*/*v*) affording 648 mg (62%, 3.31 mmol) of a faint yellow oil after drying in oil pump vacuum.

Since a distillation was not suitable with such a small amount, high vacuum was used to remove residual solvents from the product. However, this diminished the yield owing to the volatile nature of the title compound. However, we gave the isolation of most pure material priority, since it was employed as reference in the following NMR-experiments. ^1^H-NMR (400 MHz, 298 K, CDCl_3_, δ in ppm): 1.26 (s, 9H), 3.81 (s, 3H), 6.83–6.88 (m, 2H), 7.41–7.47 (m, 2H). ^13^C-NMR (100 MHz, 298 K, CDCl_3_, δ in ppm): 30.9, 45.6, 55.4, 114.1, 123.7, 139.0, 160.3. MS (EI+, *m*/*z*, [M]^+^): calc. for C_11_H_16_OS, 196.0922; found 196.0917.

1,2,4,5-Tetrakis(*tert*-butylthio)benzene **5:** 1,2,4,5-Tetrabromobenzene (10.0 g, 25.4 mmol), S-*tert*-butyl isothiouronium bromide (26.0 g, 122 mmol, 4.80 eq.), potassium *tert*-butoxide (45.6 g, 406 mmol, 16.0 eq.), tris(dibenzylideneacetone)dipalladium(0) (0.582 g, 0.63 mmol, 2.5 mol%) and triphenylphosphine (0.666 g, 2.54 mmol, 10 mol%) are placed in a Schlenk tube under argon atmosphere and are subsequently dissolved in dry DMF (200 mL). The mixture is then stirred for 16 h at 100 °C.

After completion of the reaction the mixture is quenched with water (200 mL) and extracted with DCM (3 × 50 mL). The organic phase is then separated, washed a few times with 2M hydrochloric acid and dried over MgSO_4_. The solvent is removed under reduced pressure and the crude product is washed with methanol. The product is obtained as an off white solid (9.63 g, 22.4 mmol, 88%). ^1^H-NMR (400 MHz, 298 K, CDCl_3_, δ in ppm): 1.36 (s, 36H), 7.94 (s, 2H). ^13^C-NMR (100 MHz, 298 K, CDCl_3_, δ in ppm): 31.5, 48.3, 139.5, 144.9. MS (EI+, *m*/*z*, [M]^+^): calc. for C_22_H_38_S_4_, 430.1856; found 430.1855.

For measuring the conversion via quantitative ^1^H-NMR, mesitylene was used as an internal standard. The above mentioned reaction was carried out using 100.7 mg 1,2,4,5-Tetrabromobenzene (255.8 µmol) with analogous stoichiometry as above and 32.2 µL mesitylene (27.9 mg, 232.5 µmol, 0.909 eq.) were added. The work-up was carried out as described and solvents were only removed with a vacuum above 600 mbar to avoid evaporation of mesitylene. The conversion was estimated to 99.6 ± 4 % based on two independent samples and integration of the ^1^H-NMR spectrum (Figure S6). The error of app. 4 % accounts for weighting uncertainty and variance between both samples.

2,2,6,6-Tetramethylbenzo[1,2-d:4,5-d′]bis[1,3]dithiole **1a:** 1,2,4,5-Tetrakis(*tert*-butylthio)benzene (1.00 g, 2.32 mmol), scandium triflate (0.114 g, 0.23 mmol, 10 mol%), and acetone (1.72 mL, 1.34 g, 23.2 mmol, 10.0 eq.) were dissolved in 10 mL dry toluene and heated to 100 °C under argon for 16 h. After reaching room temperature, 10 mL 0.5 M HCl was added to the reaction mixture and the organic phase was separated off. After washing the aqueous phase was 10 mL diethyl ether, the unified organic phases were dried over MgSO_4_ and solvents were removed under reduced pressure. The title compound was obtained as a pure white solid in a yield of 0.546 g (82%, 1.90 mmol). ^1^H-NMR (400 MHz, 298 K, CDCl_3_, δ in ppm): 1.88 (s, 12H), 7.02 (s, 2H). ^13^C-NMR (XY MHz, 298 K, CDCl_3_, δ in ppm): 31.4, 65.9, 116.9, 135.9. MS (ESI+, *m*/*z*, [M]^+^): calc. for C_12_H_14_S_4_, 285.9978; found 285.9971.

General procedure for condition screening with 4-bromoanisole. 4-Bromoanisole (100 mg, 0.535 mmol), *tert*-butyl isothiouronium bromide (137mg, 0.641 mmol, 1.2 eq.), tris(dibenzylideneacetone)dipalladium(0) (2.5 mol%), phosphine ligand (10 mol%) and base (4.0 eq.) were dissolved in dry and degassed DMF (4 mL) under argon atmosphere. The mixture was stirred for 19 h at the given temperature. Afterwards, the reaction was quenched with water (10 mL) and extracted with DCM (3 × 3 mL). The unified organic phases were repeatedly washed with 2M-HCl-solution (3 × 5 mL) and dried over MgSO_4_. The solvents were removed under reduced pressure regularly giving an oil containing rests of DMF. Due to the volatile nature of the products, drying of the resulting oil in high vacuum was not performed.

The analysis of the crude products was performed by ^1^H-NMR in CDCl_3_ and independently prepared samples of 4-bromoanisole, 4-bromophenol, and 4-methoxy-*tert*-butylthiobenzene (*vide infra*) were used as external reference as shown in Figure S15. It should be noted, that the chemical shifts appeared sensitive to traces of DMF remaining in the crude products, so that the reference spectra were recorded in CDCl_3_ containing 1% DMF.

General procedure for screening with other substrates. The respective educt (100 mg), S-*tert*-butylisothiouronium bromide (1.20 eq.), tris(dibenzylideneacetone)dipalladium(0) (2.5 mol%), triphenyl phosphine (10 mol%), and potassium *tert-*butanolate (4.0 eq.) were placed in a Schlenk tube and dissolved in 4 mL dry DMF. The reactions were stirred at the indicated reaction temperature and afterwards quenched by addition of 10 mL water and extracted with DCM (3 × 3 mL). The unified organic phases were repeatedly washed with 2M-HCl-solution (3 × 5 mL) and dried over MgSO_4_. The solvents were removed under reduced pressure regularly giving an oil containing rests of DMF. Due to the volatile nature of the products, drying of the resulting oil in high vacuum was not performed.

## 4. Conclusions

In this work, odorless S-*tert*-butylisothiouronium bromide is exploited as a surrogate for *tert*-butyl thiol for C-S cross coupling reactions yielding *tert*-butyl aryl thioethers. Interestingly, the ligand performance dropped with increasing steric congestions, though sterically demanding ligands are known to accelerate the often rate-determining reductive elimination. Therefore, the nucleophilic displacement step is assumed to be rate-determining in the present case. Accordingly, simple Ph_3_P yields quantitative conversions, while more sophisticated ligands, including those of the biarylphosphine type, failed to provide sufficient reactivity. While the reaction conditions can be tuned even milder with aryl iodides, the presented procedure cannot be expanded to chloro- and fluoroarenes. With activated nitroarenes, however, thioetherification already occurs at room temperate but via an S_N_Ar reaction instead of a catalytic cross-coupling process. Based on this, efficient access to benzo[1,2-d;4,5-d′]bis[1,3]dithioles via C-S cross coupling of 1,2,4,5-tetrabromobenzene is established, outperforming the previous route via S_N_Ar-reactions. Additionally, the ease of thioketal formation was enhanced by using catalytic amounts of Sc(OTf)_3_ instead of stoichiometric amounts of less effective and hazardous Lewis-acids.

## Figures and Tables

**Table 1:** Screening of various phosphine ligands for C-S cross coupling to **7**.

No.	T [°C]	Ligand	Conversion * [%]	7 * [%]	8 * [%]	Selectivity to 7 [%]
1	50	Ph_3_P	23	14	9	59
2	50	XPhos	7	0	7	0
3	50	XantPhos	0	0	0	0
4	80	Ph_3_P	100	100	0	100
5	80	dppf	69	34	35	49
6	80	XantPhos	38	2	36	4.8
7	80	XPhos	43	0	43	0
8	80	BrettPhos	45	0	45	0
9	80	SPhos	62	0	62	0
10	80	^n^Bu_3_P	78	61	17	78
11	80	none	59	0	59	0
12 **	80	none	72	0	72	0
13 **	80	SPhos	65	0	65	0

* ratios estimated from ^1^H-NMR of the crude reaction mixtures, signal assignment according to independently prepared samples of 7 and 8 (SI). ** without Pd_2_dba_3_.

**Table 2:** Screening of bases and solvent.

No.	Base	Solvent	Conversion * [%]	7 * [%]	8 * [%]	Selectivity to 7 [%]
1	KO*^t^*Bu	DMF	100	100	0	100
2	KO*^t^*Bu	*n*BuOH	61	61	0	100
3	Cs_2_CO_3_	DMF	19	19	0	100
4	K_2_CO_3_	DMF	42	42	0	100
5 **	K_2_CO_3_	DMF	0	0	0	0
6	K_3_PO_4_	DMF	43	43	0	100
7 **	K_3_PO_4_	DMF	12	12	0	100
8	KO*^t^*Bu ***	DMF	22	22	0	100

All reactions were carried out at 80 °C with 1.2 eq. **6**, 10 mol% Ph_3_P, 2.5 mol% Pd_2_dba_3_, and 4.0 eq. of base. * ratios estimated from ^1^H-NMR of the crude reaction mixture, signal assignment according to independently prepared samples of **7** and **8**. ** 10% mol% 18-crown-6 added. *** 2.4 eq. used.

**Table 3:** Screening of further substrates.

No.	Substrate	Catalyst	T [°C]	Yield * [%]
1 *	1,4-diiodobenzene	Ph_3_P/Pd_2_dba_3_	80	100
2 *	1,4-diiodobenzene	Ph_3_P/Pd_2_dba_3_	50	100
3	4-nitrobromobenzene	Ph_3_P/Pd_2_dba_3_	25	100
4	4-nitrobromobenzene	none	25	100
5 **	4-chloroansiole	Ph_3_P/Pd_2_dba_3_	80	0
6 **	4-chloroanisole	none	80	0
7 **	4-fluoroanisole	Ph_3_P/Pd_2_dba_3_	80	0
8 **	4-fluoroanisole	none	80	0

All reactions were carried out in dry DMF for 19 h with 1.2 eq. **6**, 10 mol% Ph_3_P, 2.5 mol% Pd_2_dba_3_, and 4.0 eq. of KO*^t^*Bu unless stated otherwise. Yields were determined from ^1^H-NMR spectra of the crude reaction mixtures. * stoichiometry was adapted to a twofold substitution accordingly. ** extensive O-demethylation was observed as outlined in the Supporting Information.

## Supplementary Materials

The following are available online. NMR-spectra and mass spectrometric data can be found in the Supporting Information.
