# Supplementary material for: Improved, Odorless Access to Benzo[1,2-d;4,5-d′]- bis[1,3]dithioles and Tert-butyl Arylsulfides via C-S Cross Coupling
Source: Molecules. 2020 Aug 12;25(16):3666. doi: 10.3390/molecules25163666 (PMC7464442; doi:10.3390/molecules25163666)
Supplement: Supplementary file 1 [file molecules-25-03666-s001.pdf]

SUPPORTING INFORMATION

Improved odorless access to benzo[1,2-d;4,5-d']bis[1,3]dithioles and *tert*-butyl arylsulfides via C-S cross coupling

Kevin Kopp, Olav Schiemann, Nico Fleck*

Institute of Physicial and Theoretical Chemistry, University of Bonn, Wegelerstr. 12, 53115 Bonn, Germany.

*E-Mail: *fleck@pc.uni-bonn.de*

Table of Contents

1. Analytical data of products........ 3
1.1 NMR spectroscopy........ 3
1.2 Mass spectrometry........ 8
2. Condition screening........ 12
2.2 ^{1}H-NMR data........ 14
3 Literature........ 27

1. Analytical data of products

1.1 NMR spectroscopy

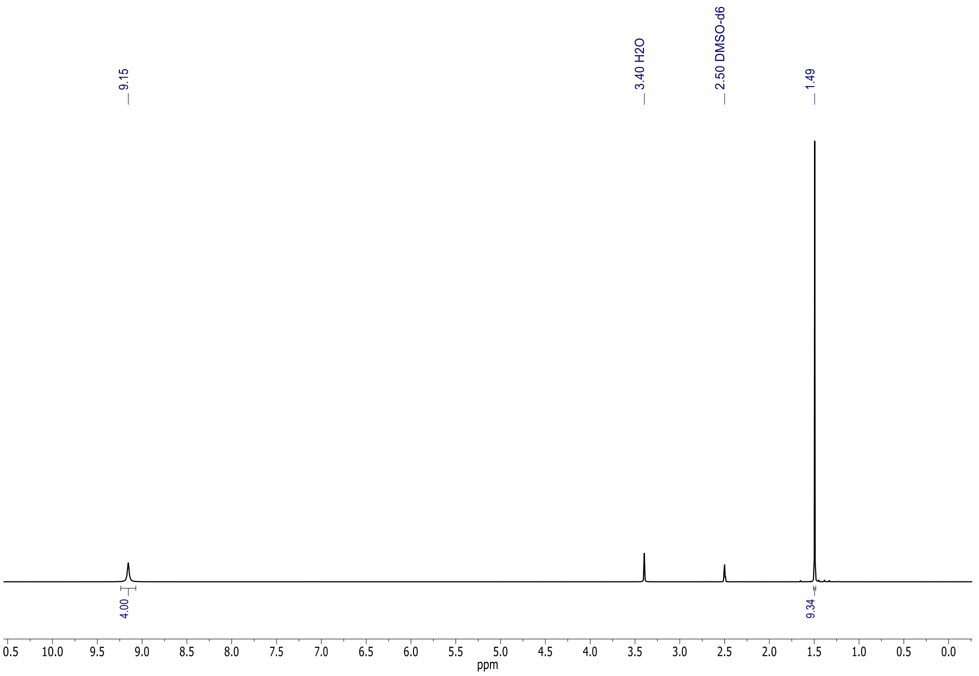

Figure S1. ^{1}H-NMR (400 MHz, 298 K, DMSO-d6) spectrum of S-*tert*-butyl isothiouronium bromide **6**.

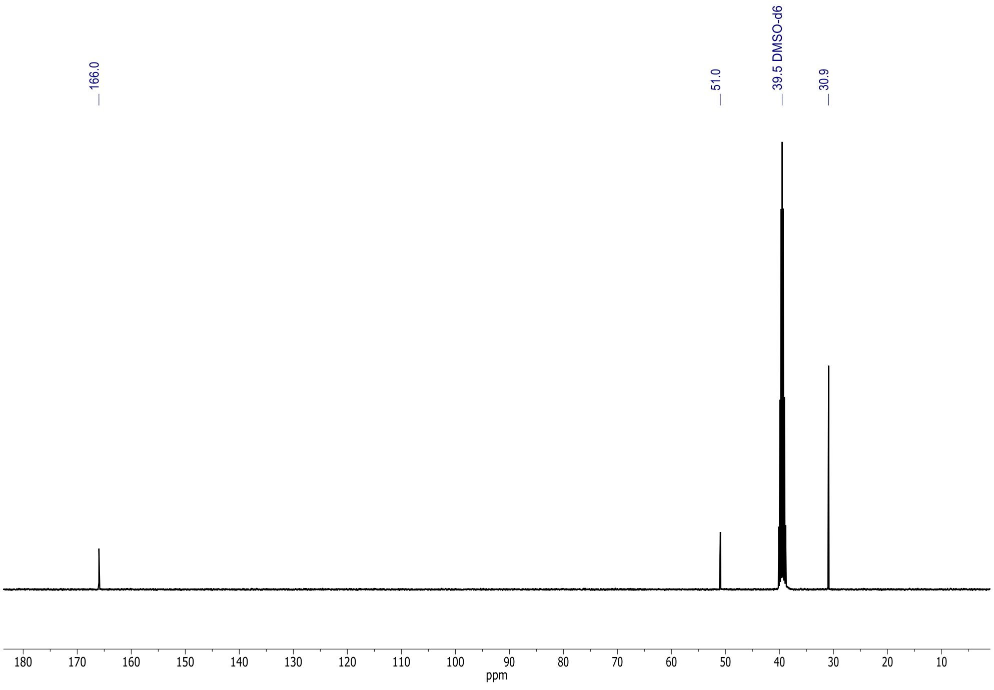

Figure S2. ^{13}C-NMR (100 MHz, 298 K, DMSO-d6) of S-tert-butyl isothiouronium bromide **6**.

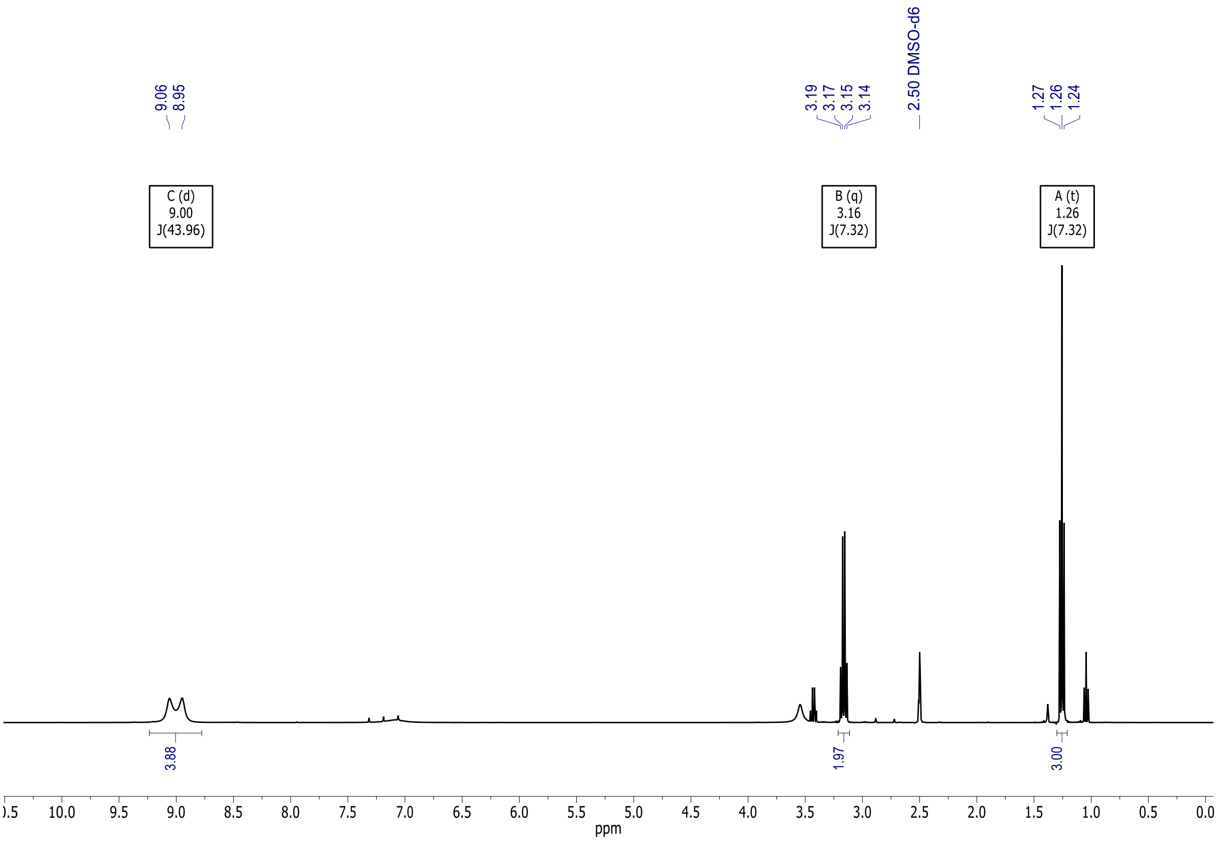

Figure S3. ^{1}H-NMR (400 MHz, 298 K, DMSO-d6) of reaction 2.1 when using EtOH as solvent.

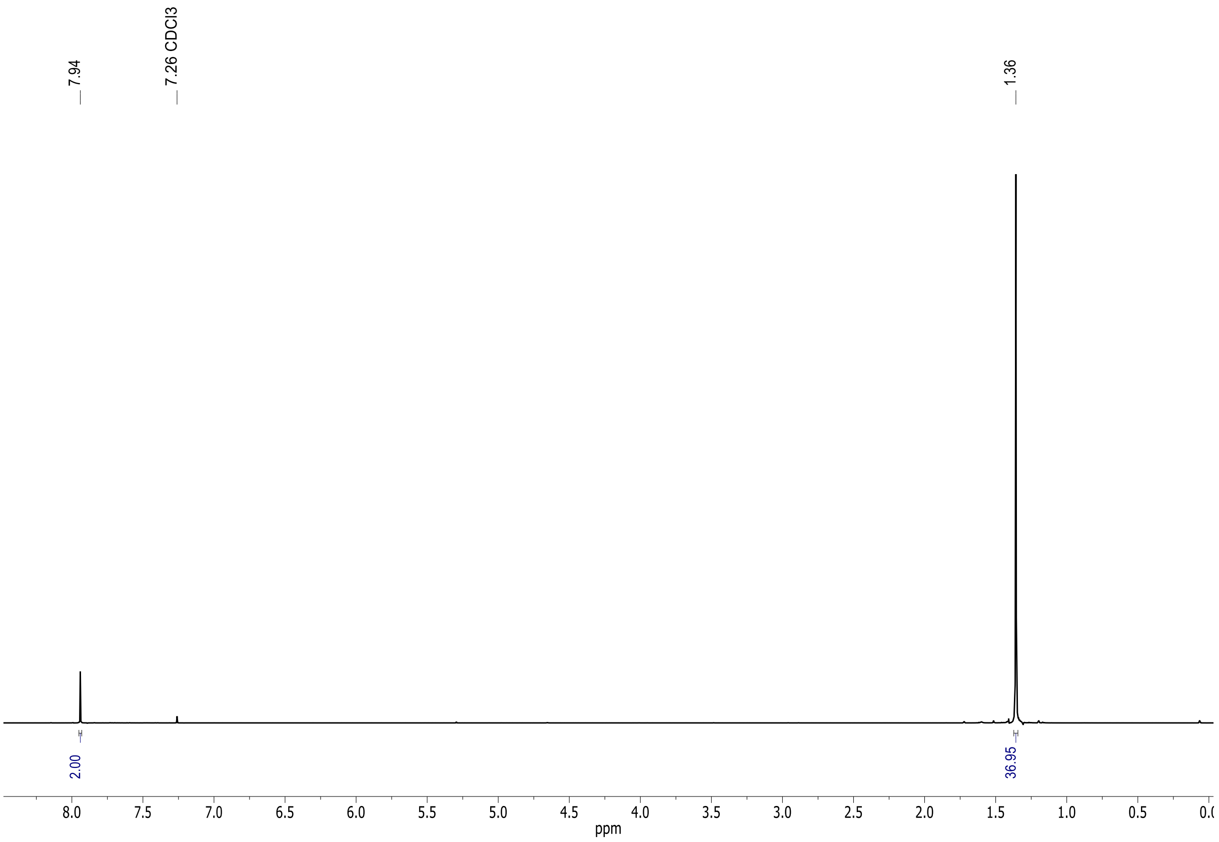

Figure S4. ^{1}H-NMR (400 MHz, 298 K, $CDCl_3$) of 1,2,4,5-Tetrakis(*tert*-butylthio)benzene **5**.

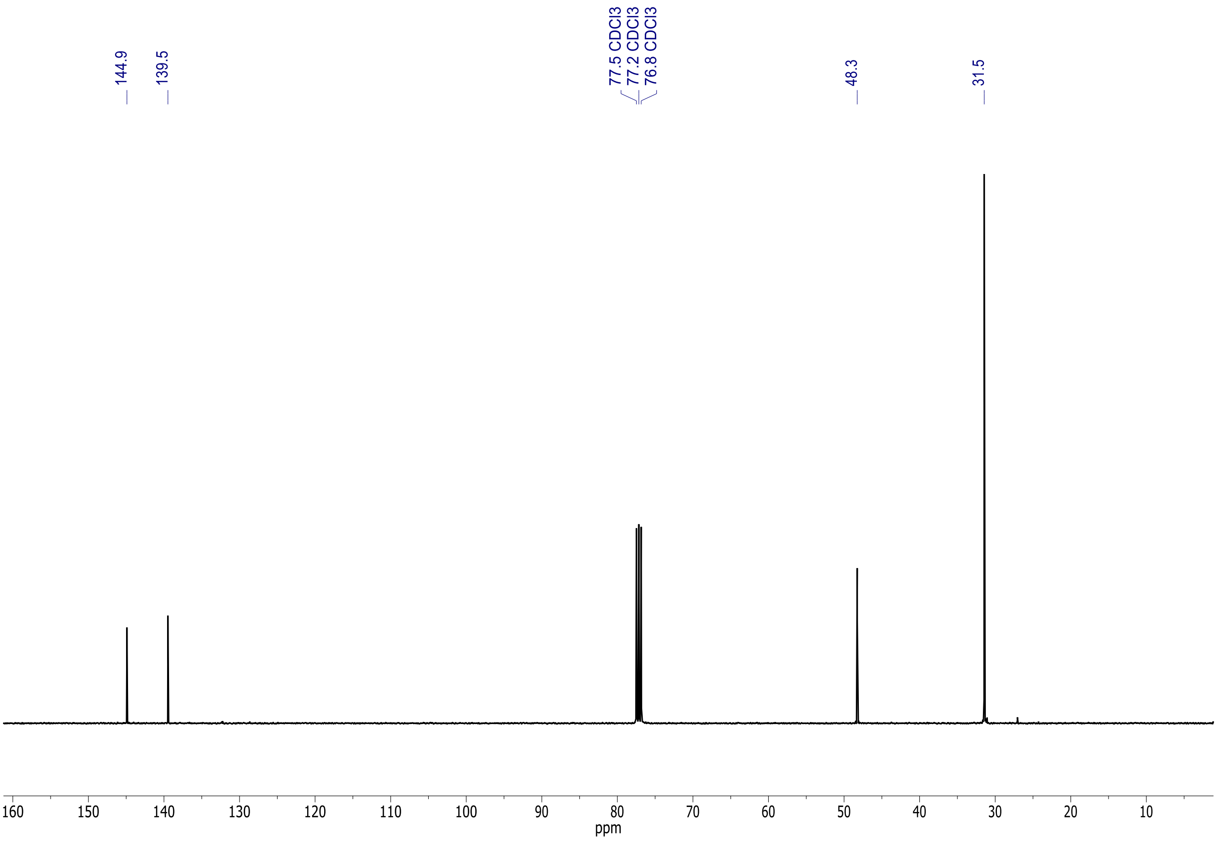

Figure S5. ^{13}C-NMR (100 MHz, 298 K, $CDCl_3$) of 1,2,4,5-Tetrakis(*tert*-butylthio)benzene **5**.

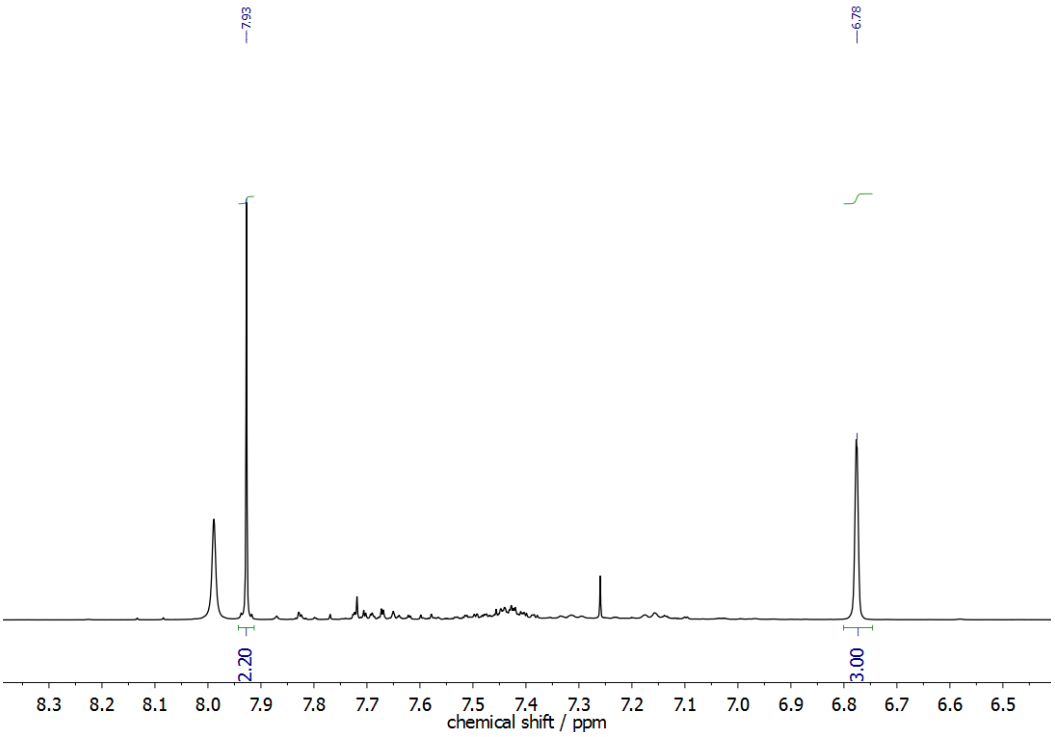

Figure S6. ^{1}H-NMR (400 MHz, 298 K, $CDCl_3$) of the reaction mixture converting 1,2,4,5-tetrabromobenzene to **5** containing mesitylene as an internal standard.

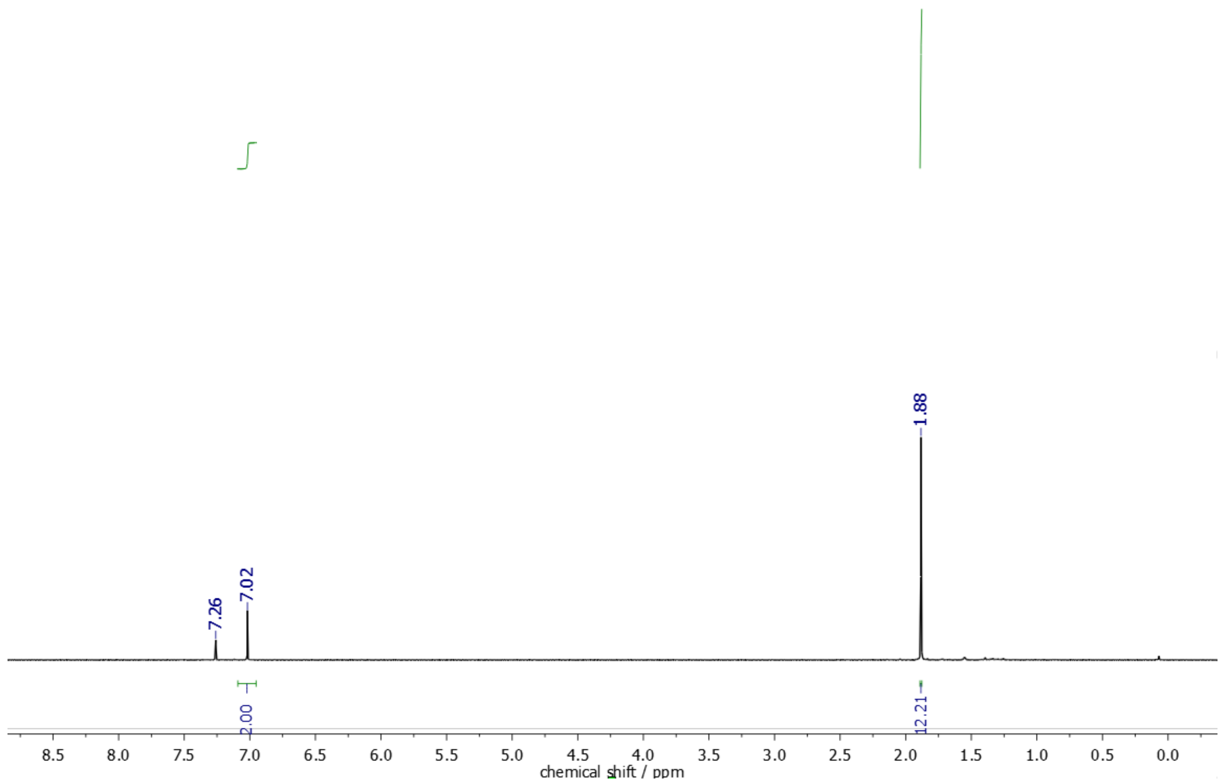

Figure S7. ^{1}H-NMR (400 MHz, 298 K, $CDCl_3$) of **1a**.

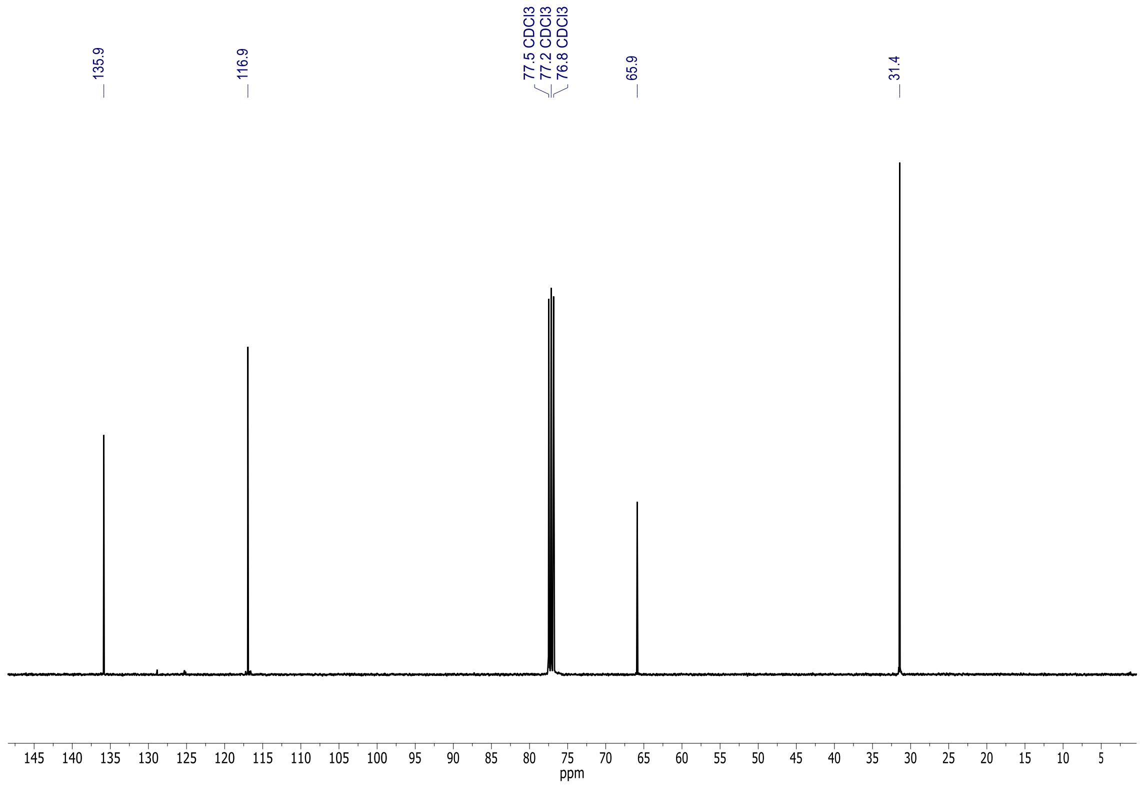

Figure S8. ^{13}C-NMR (100 MHz, 298 K, $CDCl_3$) of **1a**.

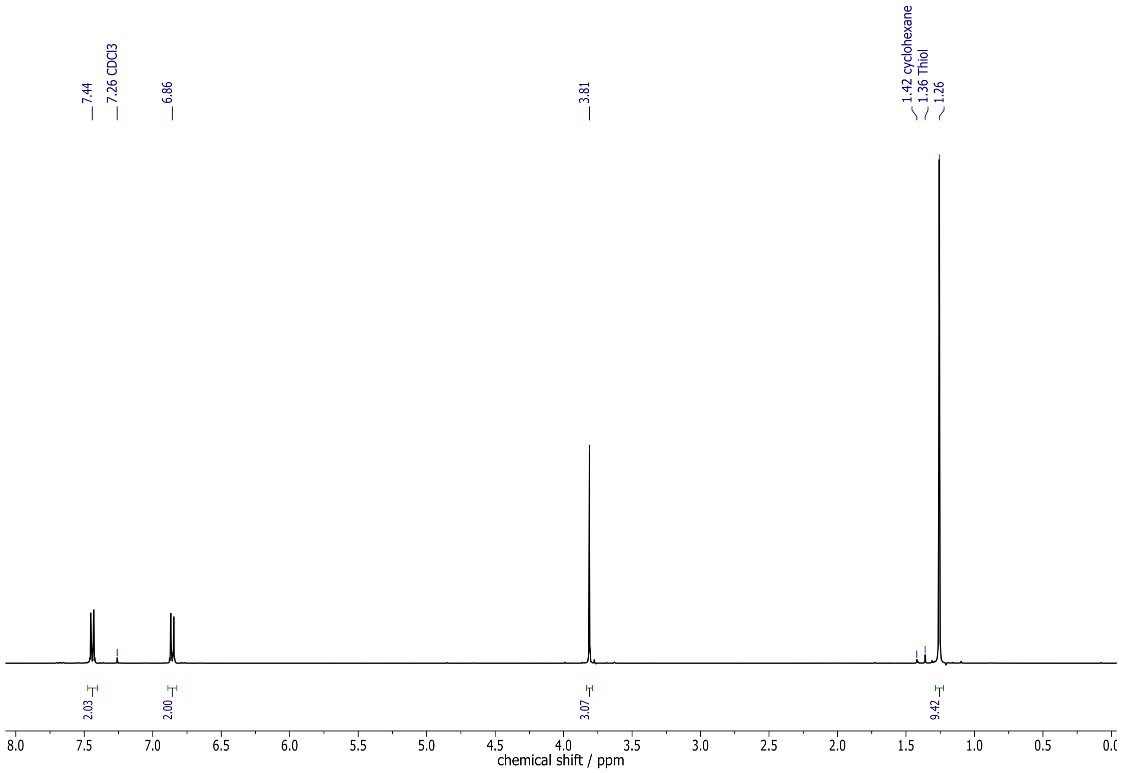

Figure S9. ^{1}H-NMR (400 MHz, 298 K, $CDCl_3$) of 4-methoxy-*tert*-butylthiobenzene **7**.

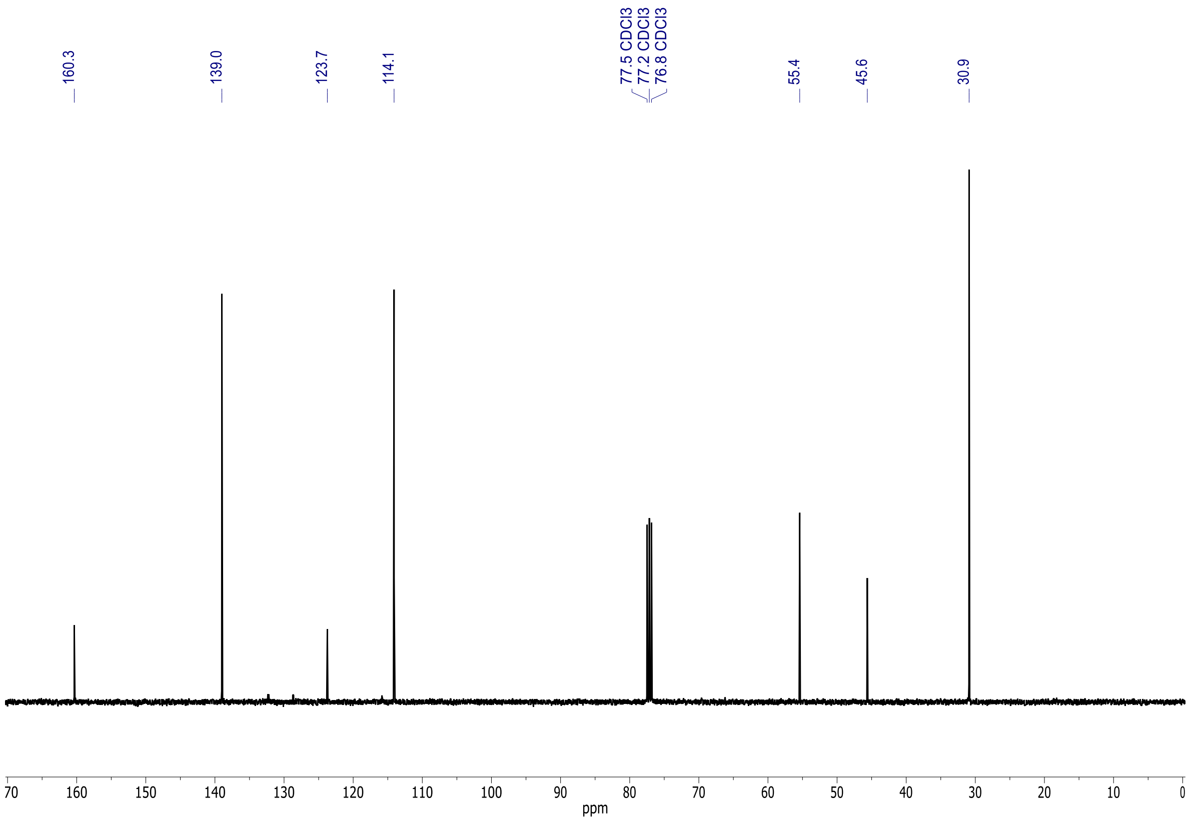

Figure S10. ^{13}C-NMR of 4-methoxy-*tert*-butylthiobenzene **7**.

1.2 Mass spectrometry

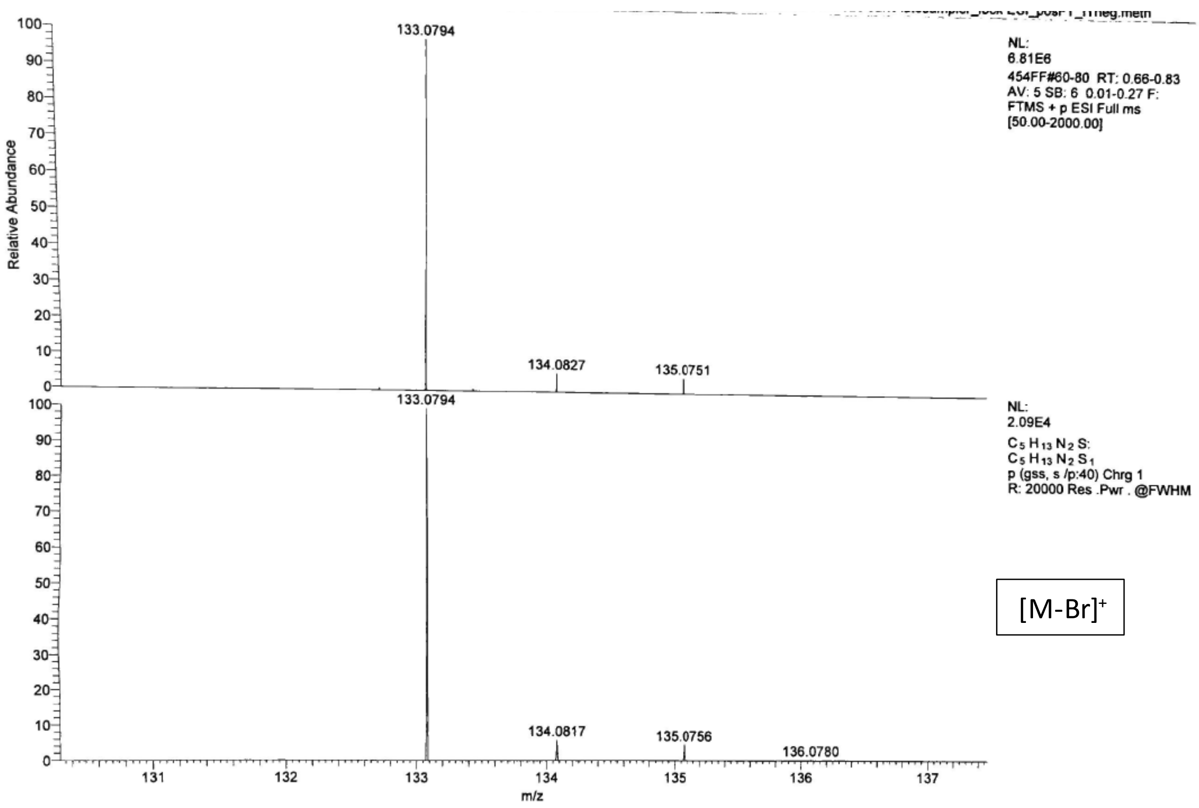

Figure S11. ESI(+)-MS (top) of S-*tert*-butyl isothiouronium bromide **6** and calculated isotope pattern (bottom).

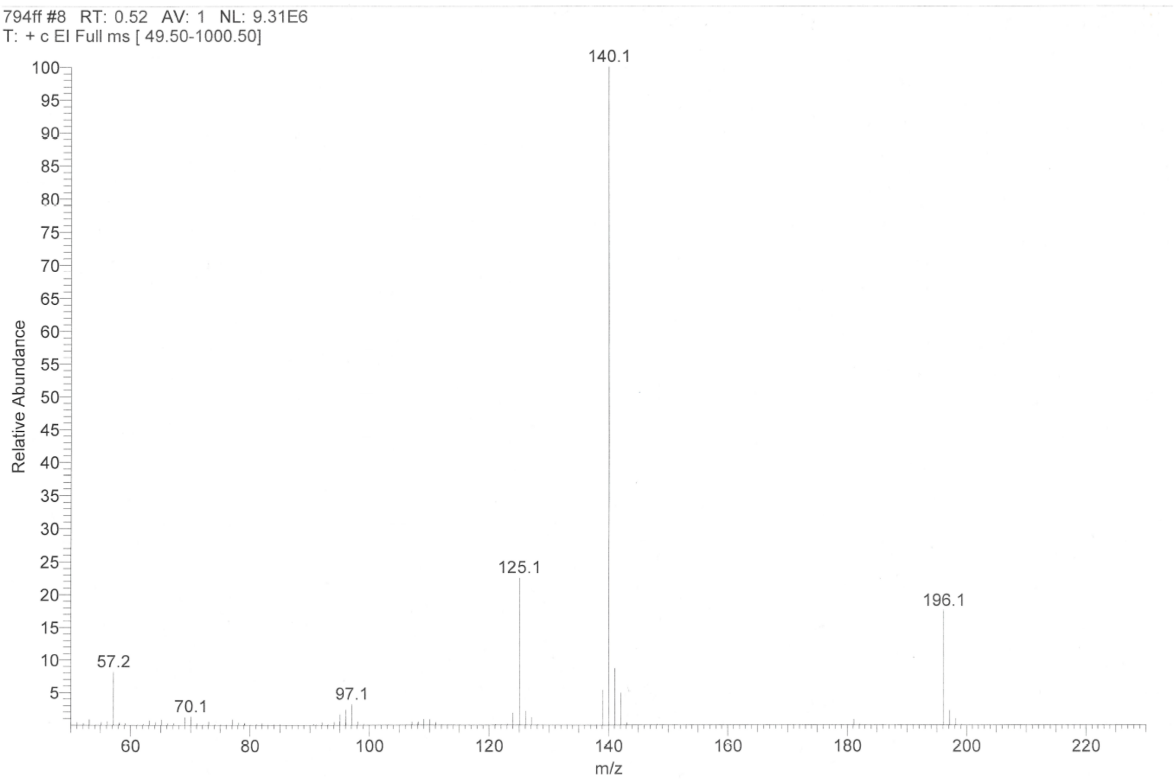

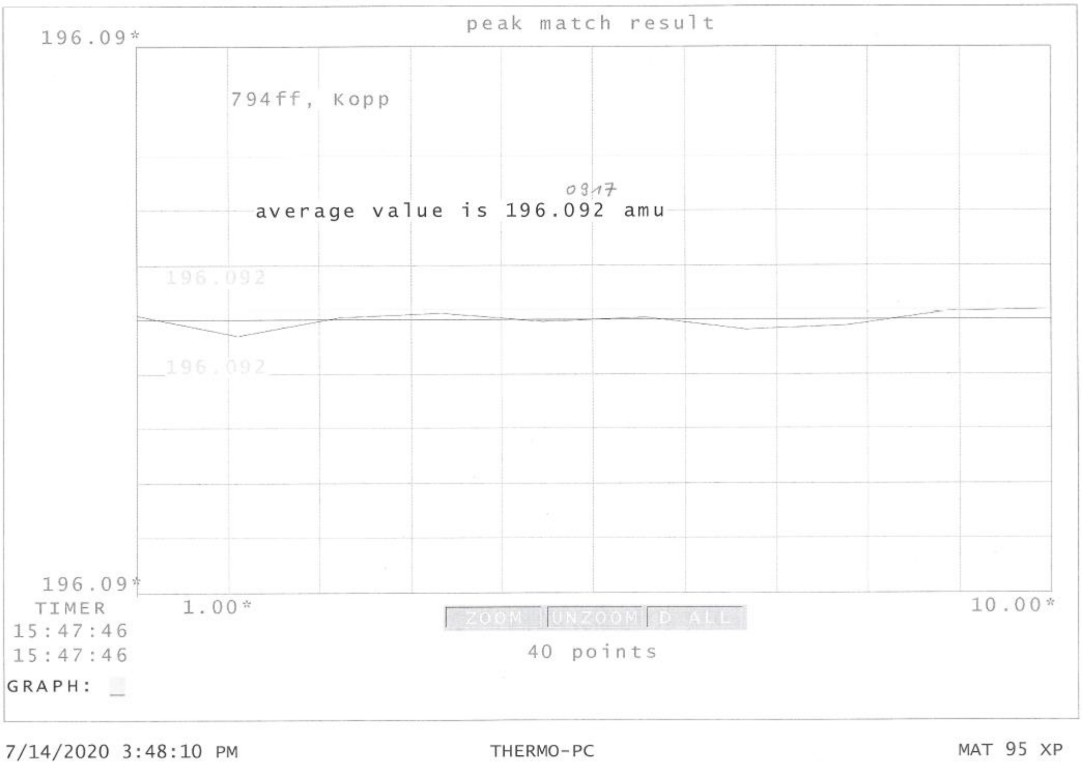

Figure S12. EI(+)-MS (top) of 4-methoxy-*tert*-butylthiobenzene **7** and average value of exact mass (bottom).

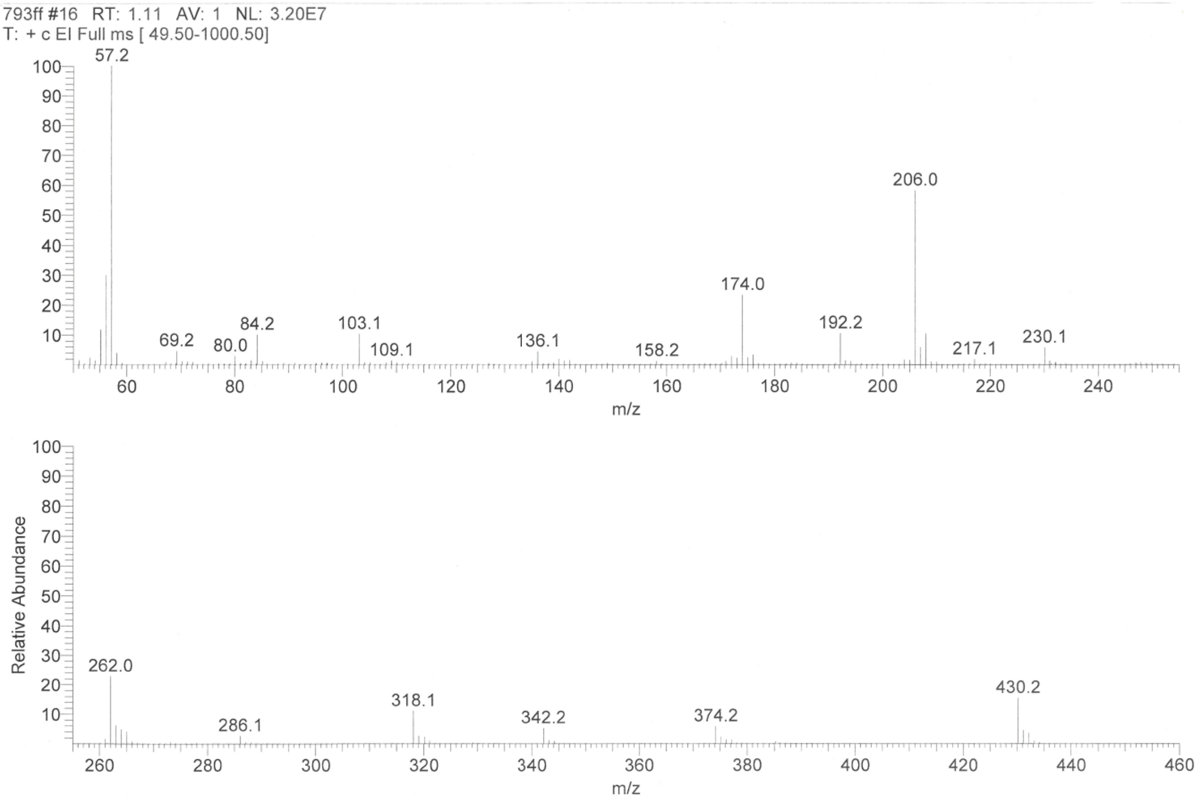

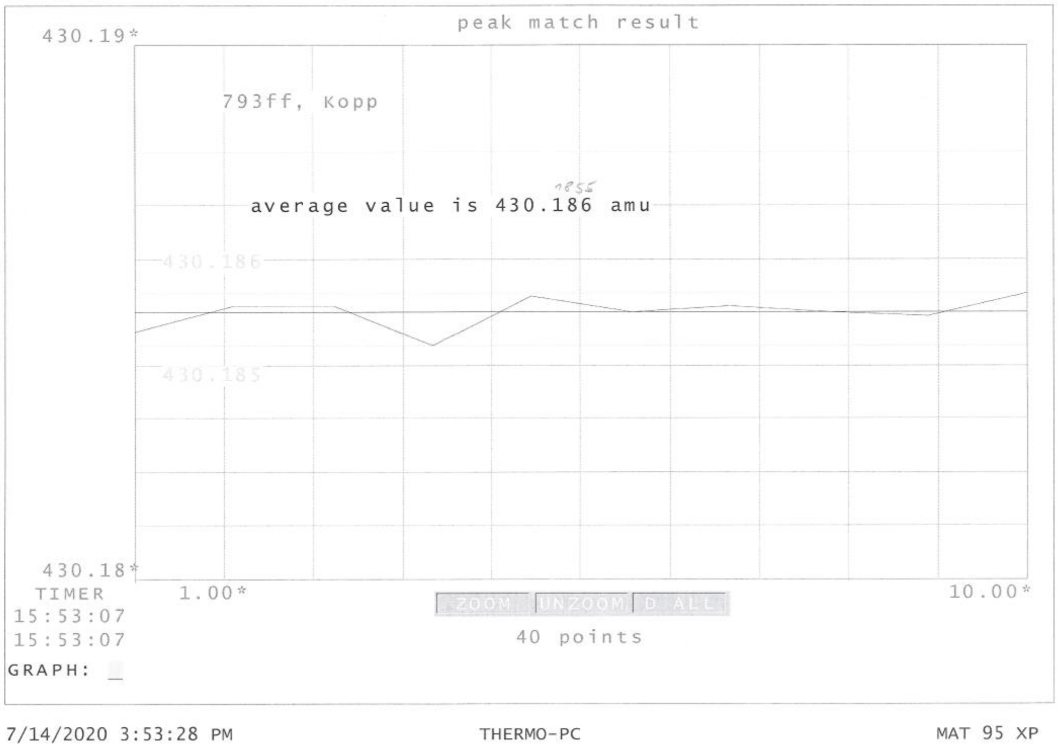

Figure S13. EI(+)-MS (top) of 1,2,4,5-tetrakis(*tert*-butylthio)benzene **5** and average value of exact mass (bottom).

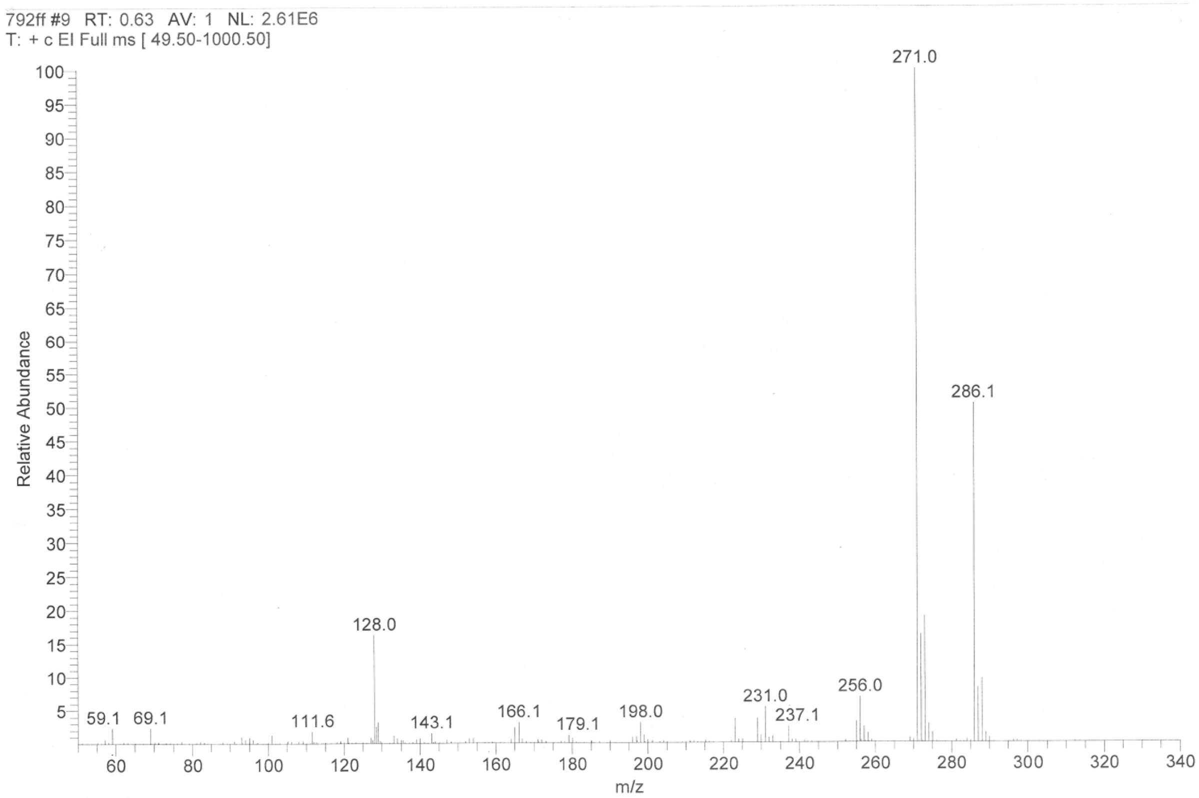

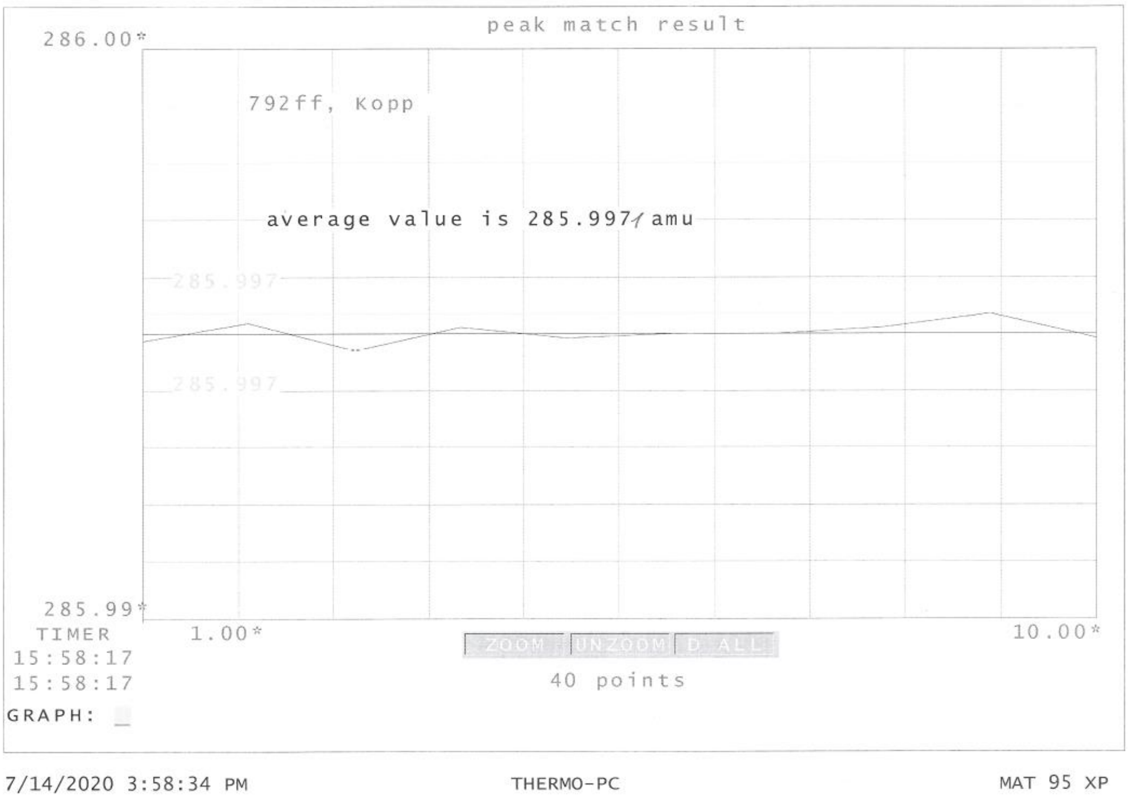

Figure S14. EI(+)-MS (top) of **1a** and average value of exact mass (bottom).

2. Condition screening

Screening of conditions with other substrates

The analysis of the reaction mixtures was carried out via ^{1}H-NMR, products were identified via comparison of literature values for 1,4-bis(*tert*-butylthio)benzene[4], 4-chlorophenol[5], 4-fluorophenol[6], and 4-(*tert*-butylthio)nitrobenzene[7].

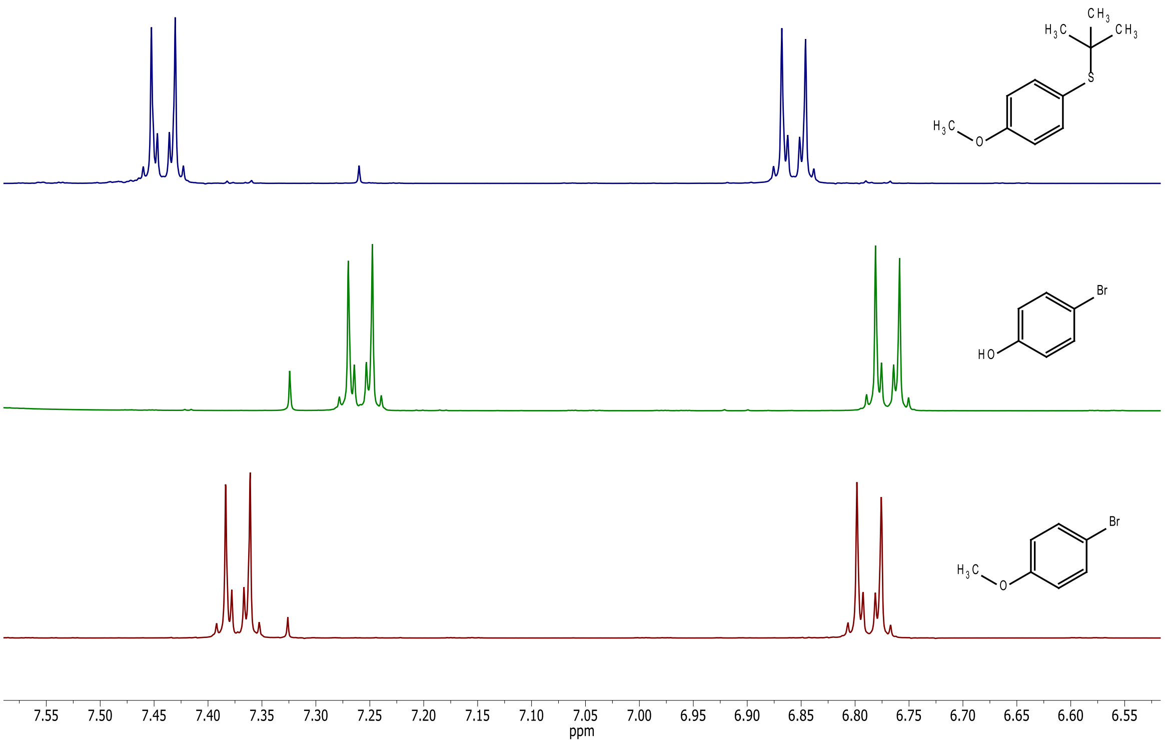

Figure S15. Reference spectra of 4-bromoanisole (bottom), 4-bromophenol (middle) and 4-methoxy-*tert*-butylthiobenzene (top).

Table S1. Reaction conditions.

Reaction No.	Temperature [°C]	ligand	base	Solvent
1	50	Ph_3P	KO^{t}Bu	DMF
2	50	XPhos	KO^{t}Bu	DMF
3	50	Xantphos	KO^{t}Bu	DMF

4	80	Ph_3P	KO^tBu	DMF
5	80	XPhos	KO^tBu	DMF
6	80	Xantphos	KO^tBu	DMF
7	80	dppf	KO^tBu	DMF
8	80	SPhos	KO^tBu	DMF
9	80	BrettPhos	KO^tBu	DMF
10	80	$^nBu_3P$	KO^tBu	DMF
11	80	none	KO^tBu	DMF
12[*]	80	none	KO^tBu	DMF
13[*]	80	SPhos	KO^tBu	DMF
14	80	Ph_3P	KO^tBu	^nBuOH
15	80	Ph_3P	K_2CO_3	DMF
16	80	Ph_3P	Cs_2CO_3	DMF
17	80	Ph_3P	K_3PO_4	DMF
18[**]	80	Ph_3P	KO^tBu	DMF
19[***]	80	Ph_3P	K_2CO_3	DMF
20[***]	80	Ph_3P	K_3PO_4	DMF

[*]without Pd_2dba_3. [**]reduced amounts of base to 2.4 eq. [***]addition of 10mol% of 18-C-6.

2.2 ^{1}H-NMR data

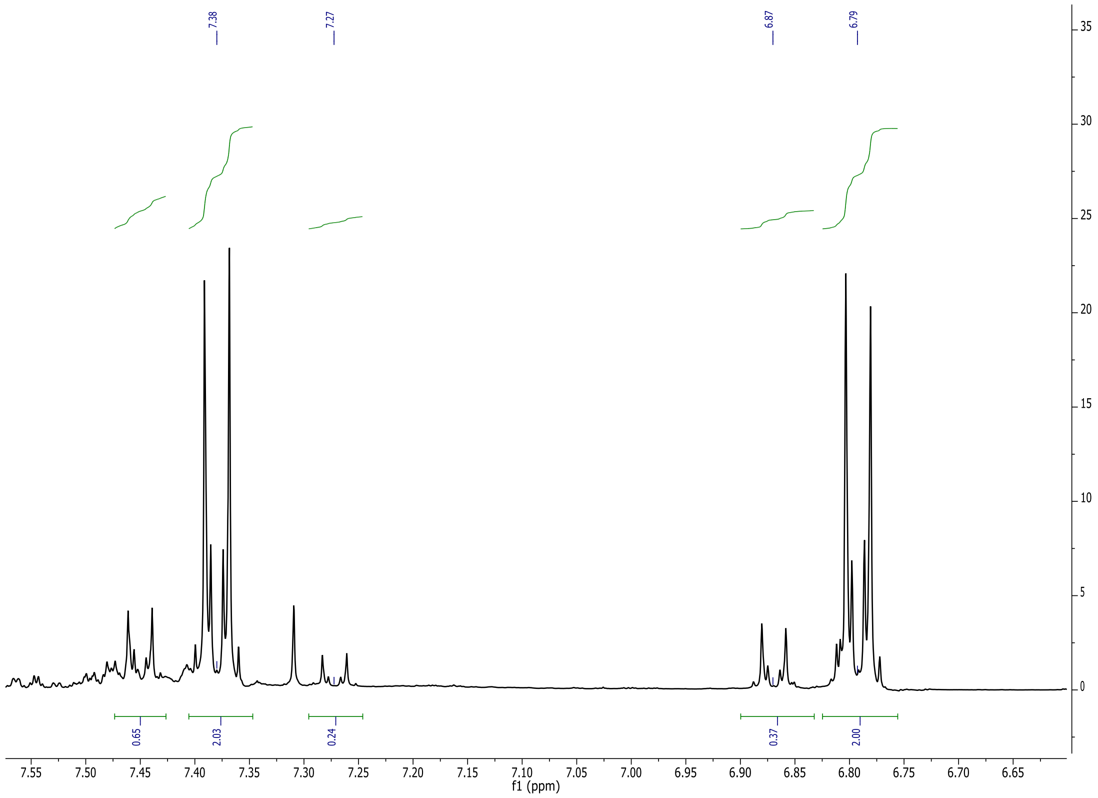

Figure S16. ^{1}H-NMR (400 MHz, 298 K, $CDCl_3$) of reaction 1.

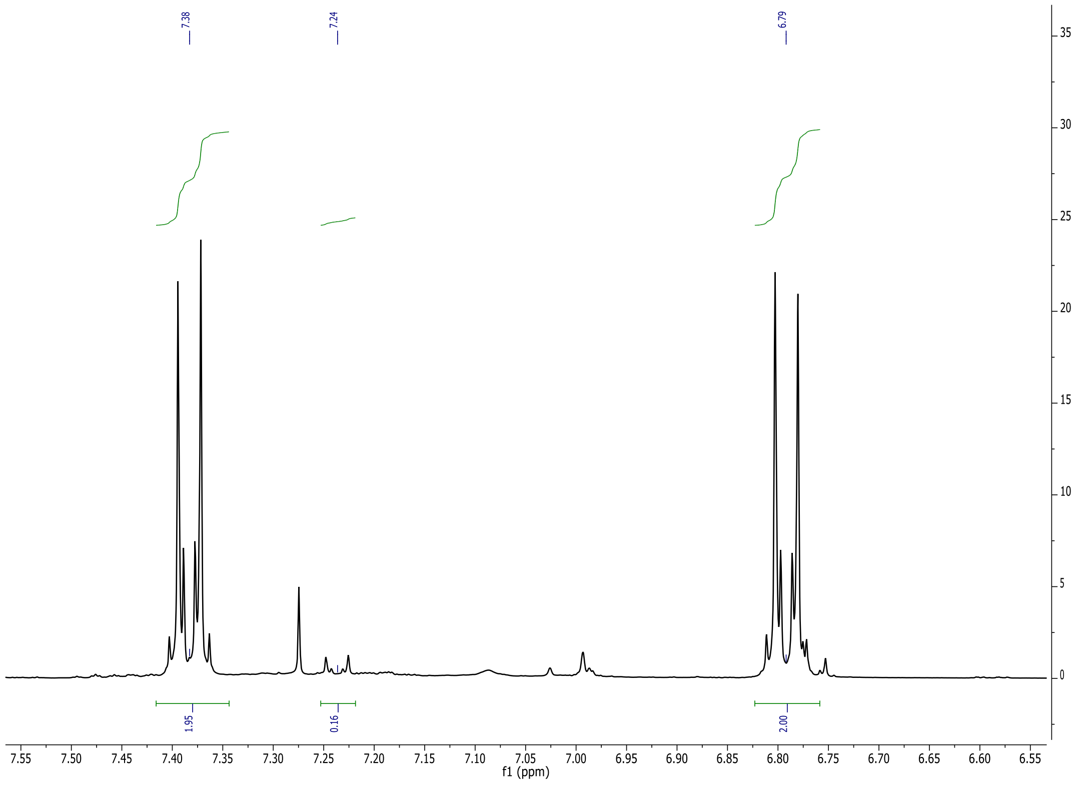

Figure S17. ^{1}H-NMR (400 MHz, 298 K, $CDCl_3$) of Reaction 2.

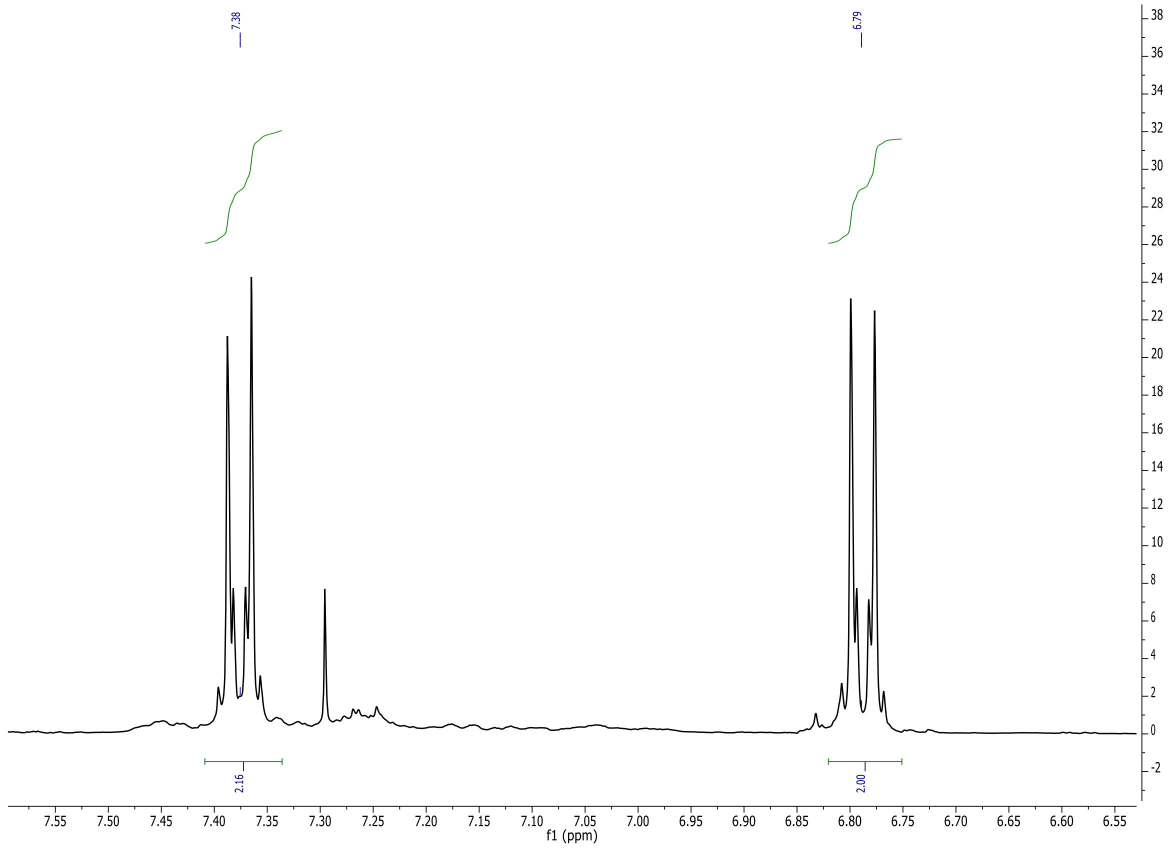

Figure S18. ^{1}H-NMR (400 MHz, 298 K, $CDCl_3$) of reaction 3.

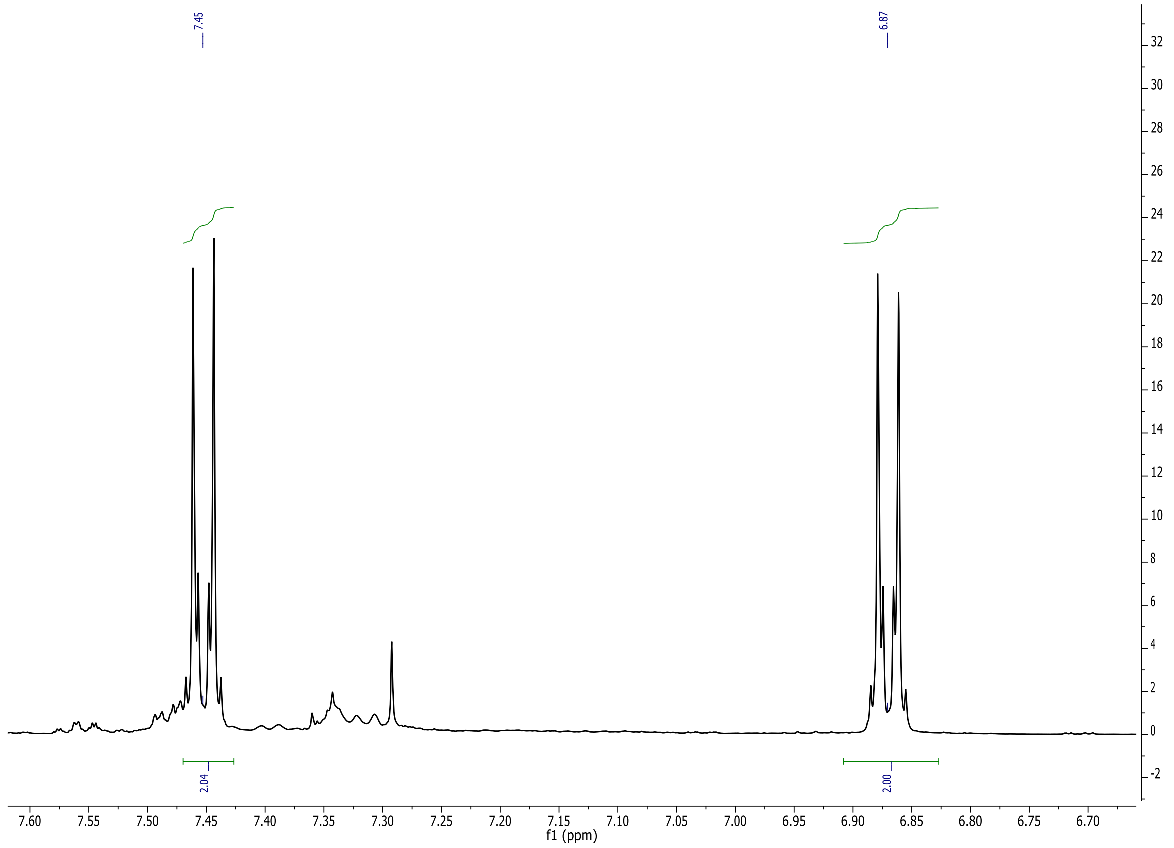

Figure S19. ^{1}H-NMR (500 MHz, 298 K, $CDCl_3$) of reaction 4.

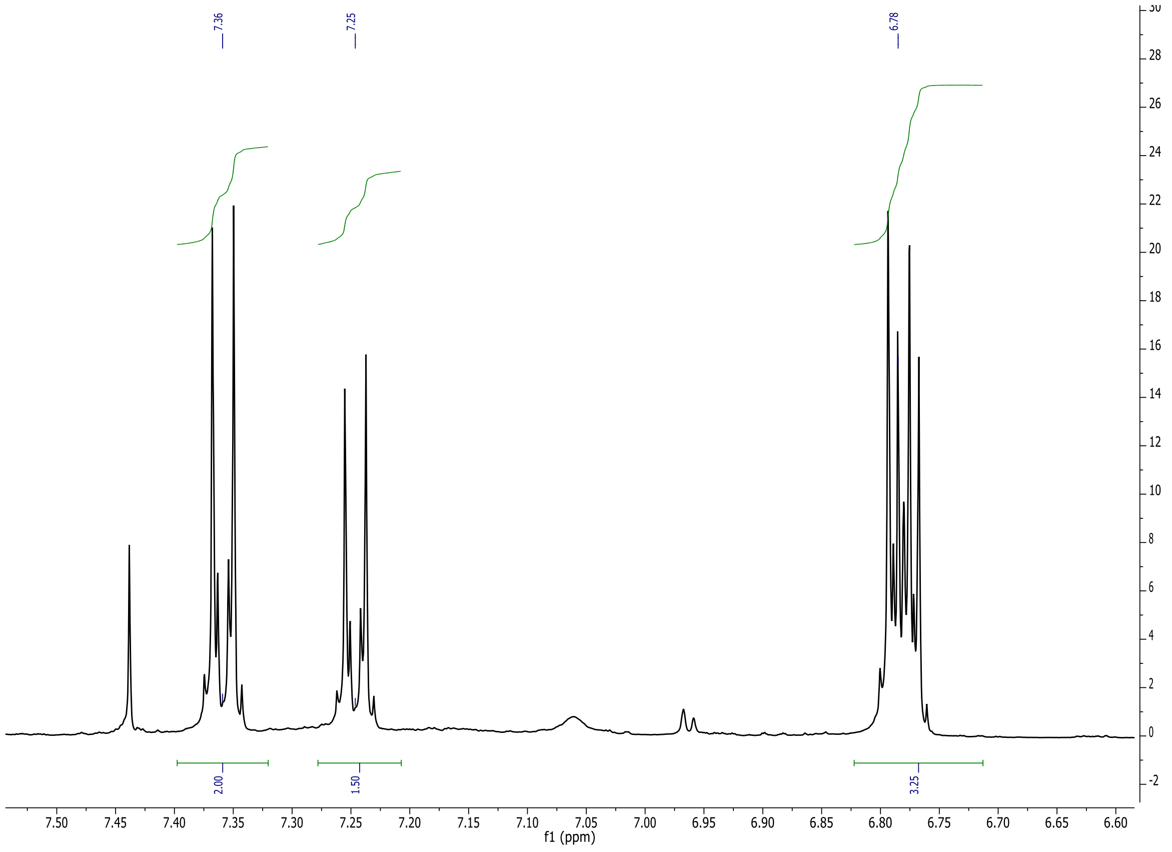

Figure S20. ^{1}H-NMR (500 MHz, 298 K, $CDCl_3$) of reaction 5.

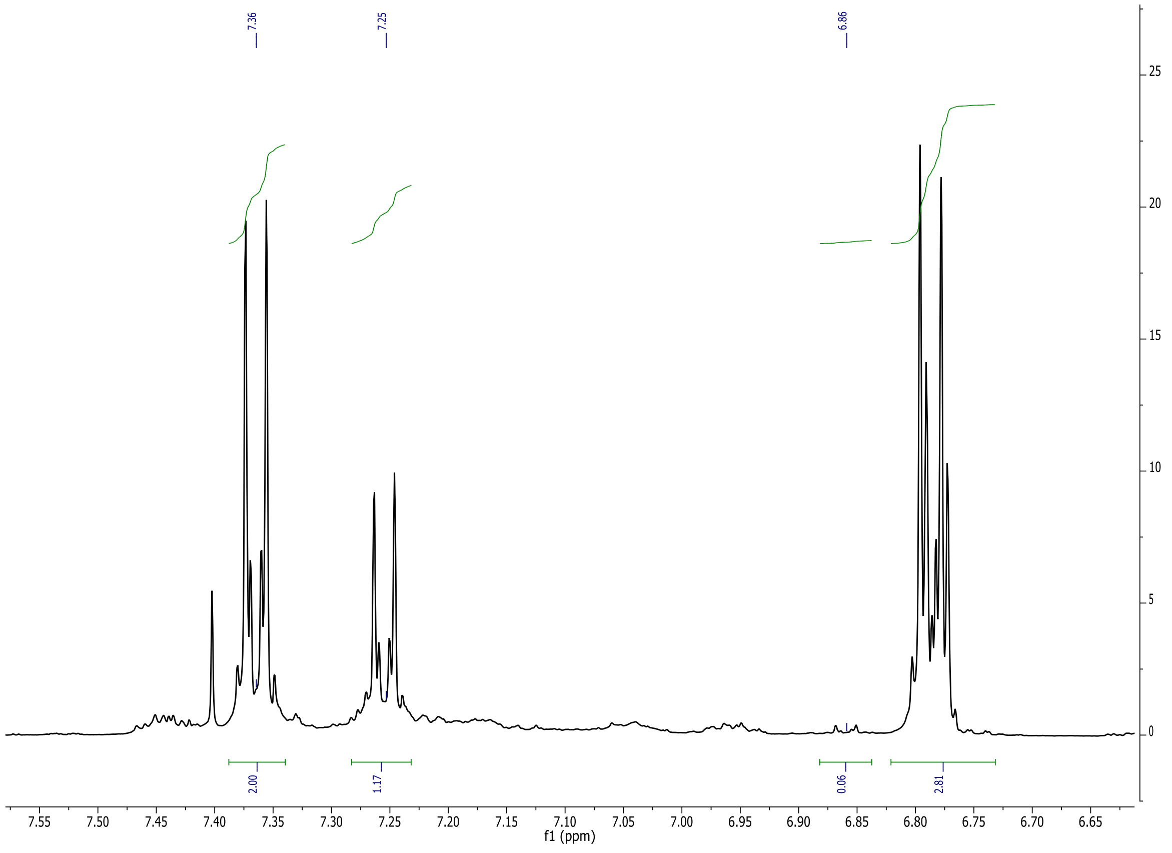

Figure S21. ^{1}H-NMR (500 MHz, 298 K, $CDCl_3$) of reaction 6.

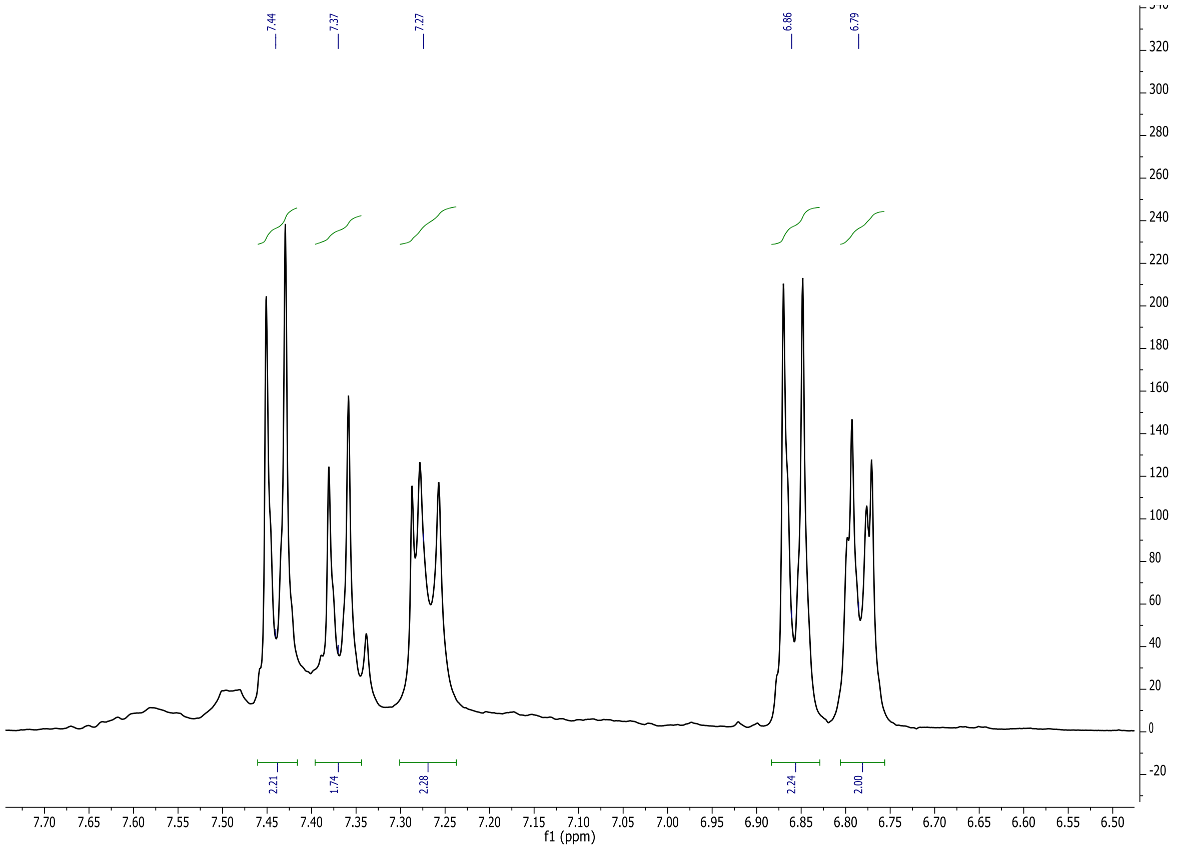

Figure S22. ^{1}H-NMR (400 MHz, 298 K, $CDCl_3$) of reaction 7.

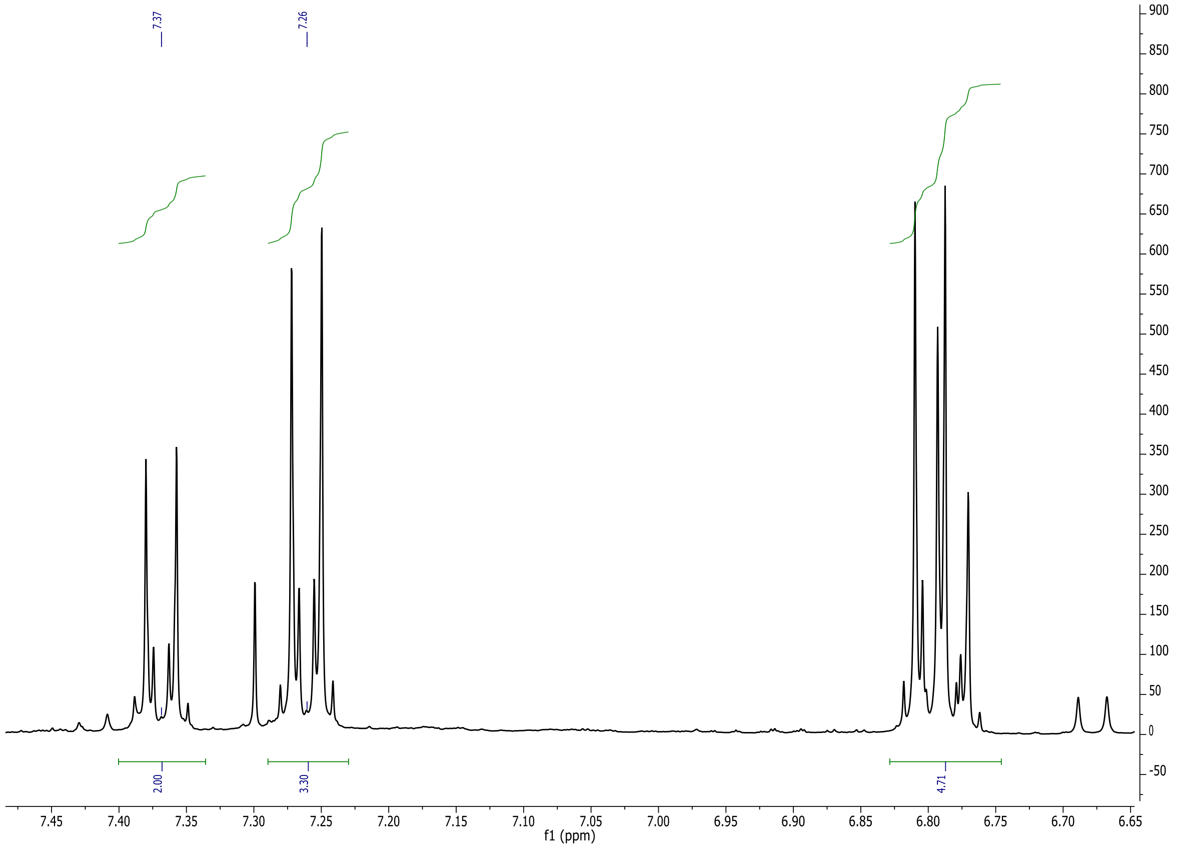

Figure S23. ^{1}H-NMR (400 MHz, 298 K, $CDCl_3$) of reaction 8.

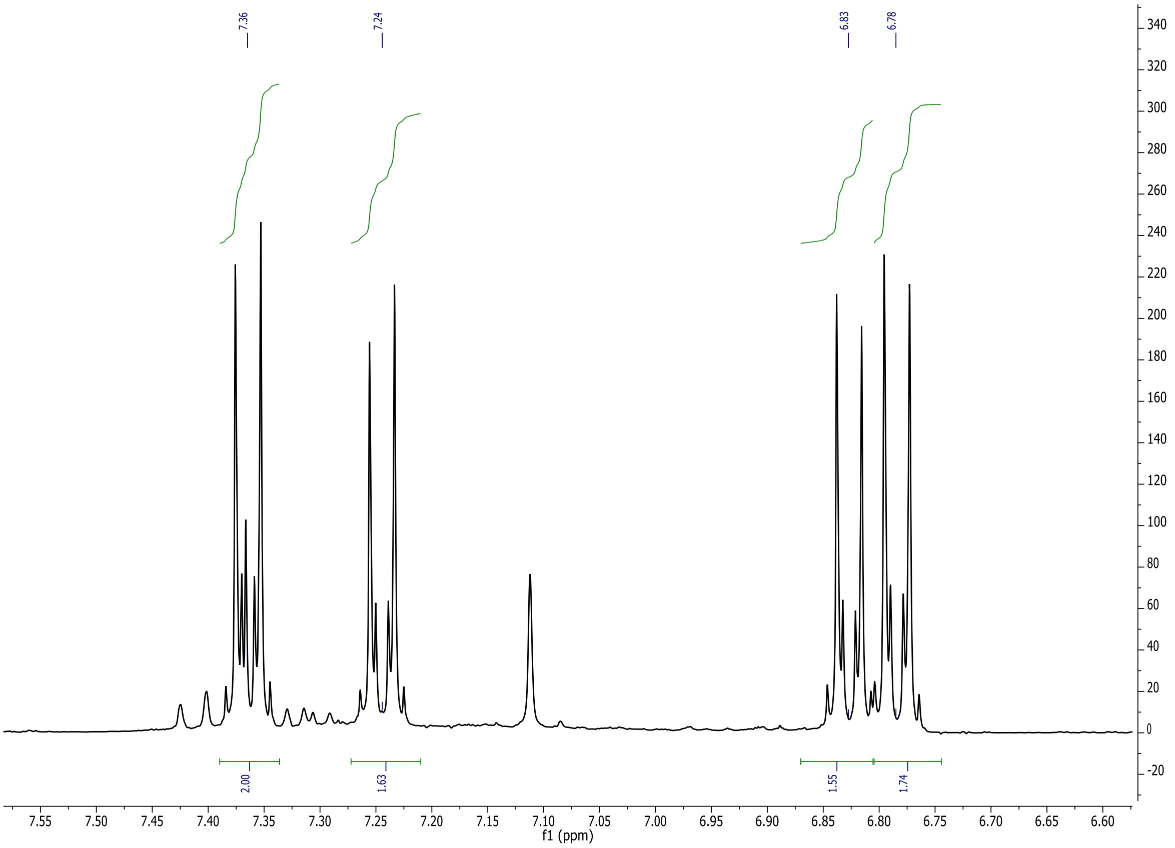

Figure S24. ^{1}H-NMR (400 MHz, 298 K, $CDCl_3$) of reaction 9.

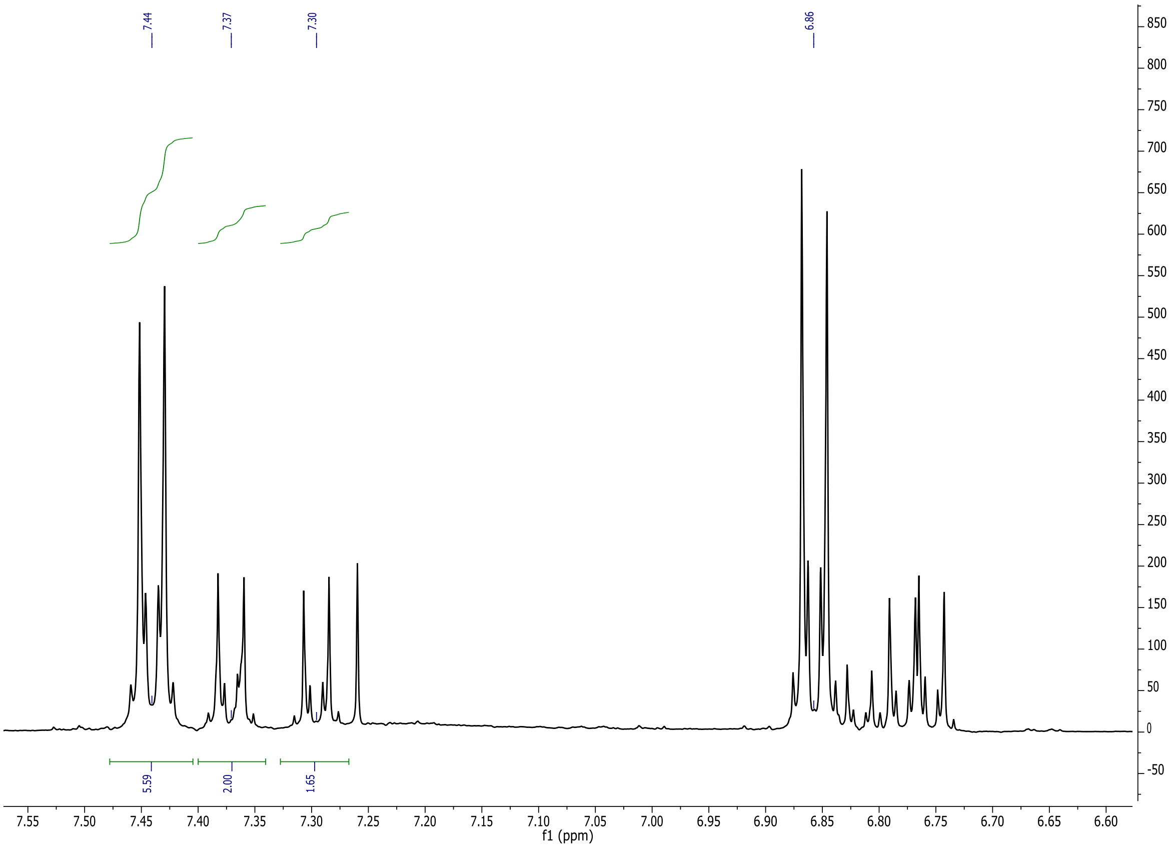

Figure S25. ^{1}H-NMR (400 MHz, 298 K, $CDCl_3$) of reaction 10.

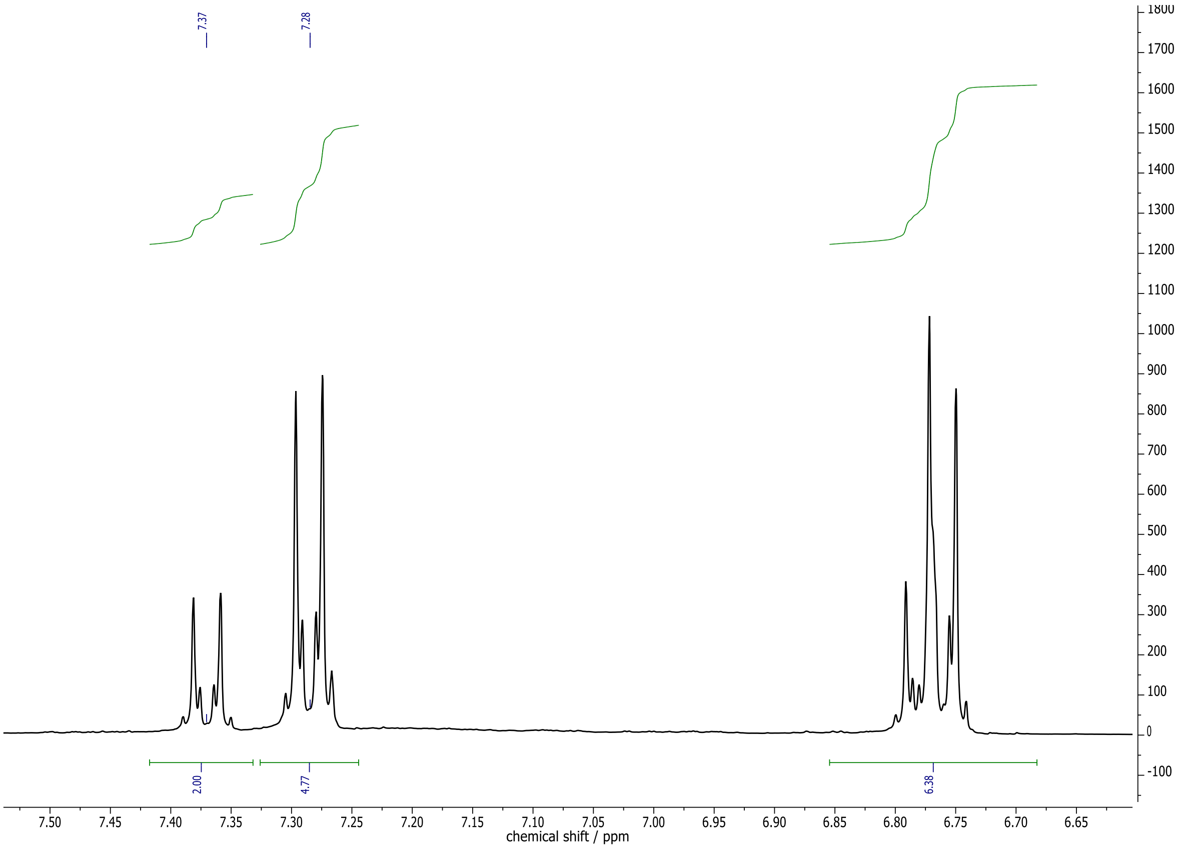

Figure S26. ^{1}H-NMR (400 MHz, 298 K, $CDCl_3$) of reaction 11.

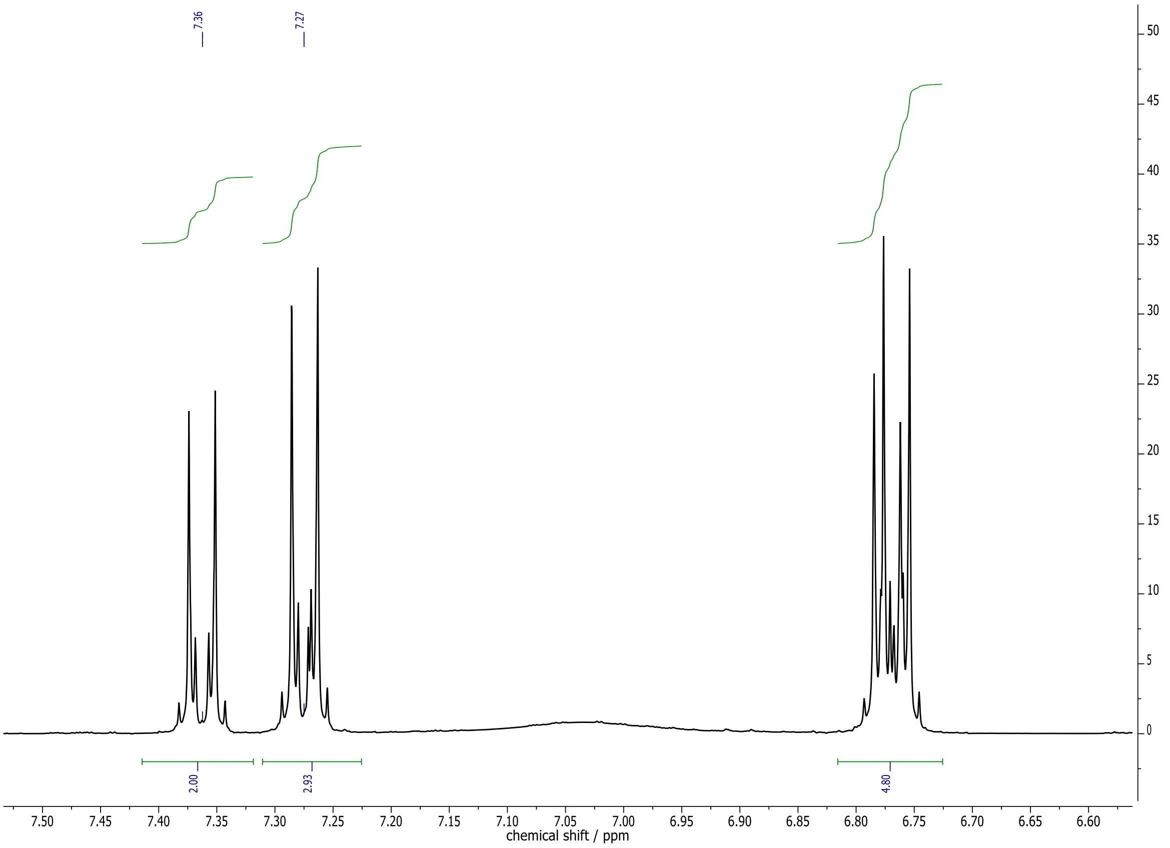

Figure S27. ^{1}H-NMR (400 MHz, 298 K, $CDCl_3$) of reaction 12.

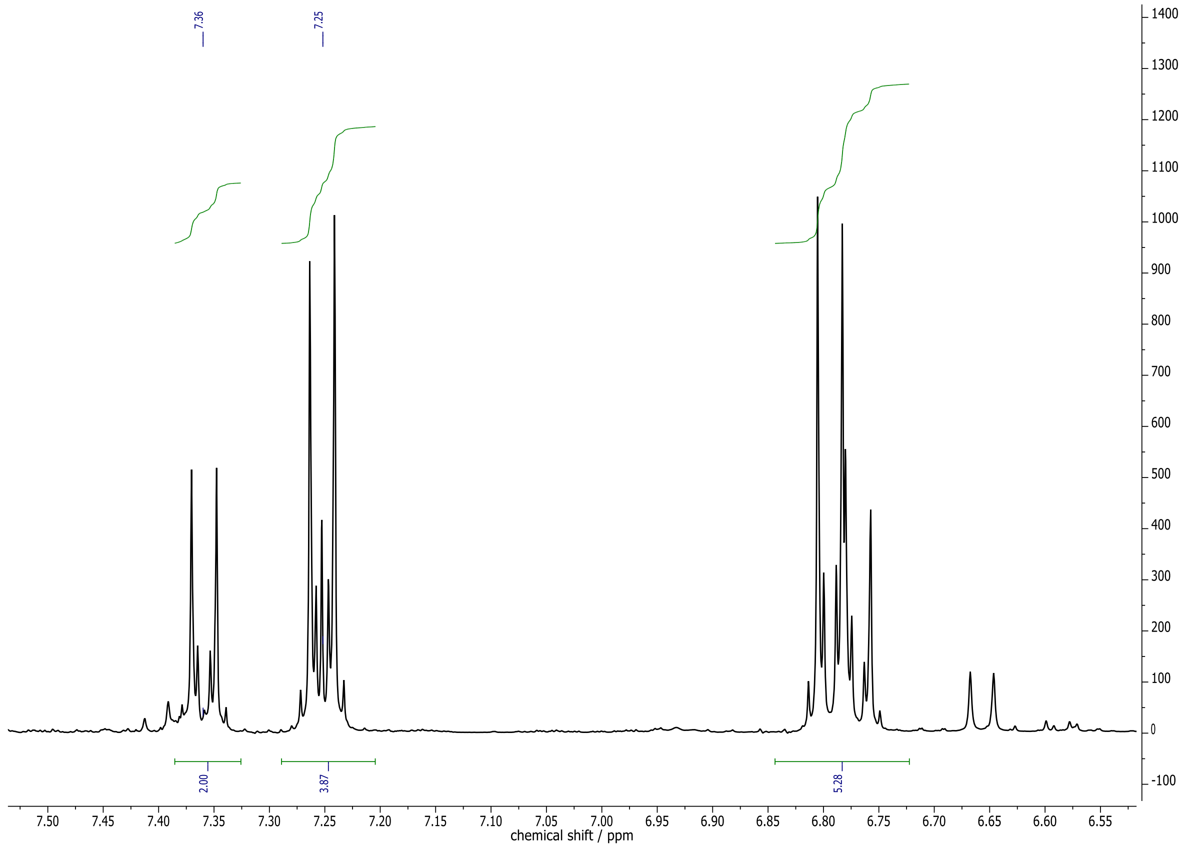

Figure S28. ^{1}H-NMR (400 MHz, 298 K, $CDCl_3$) of reaction 13.

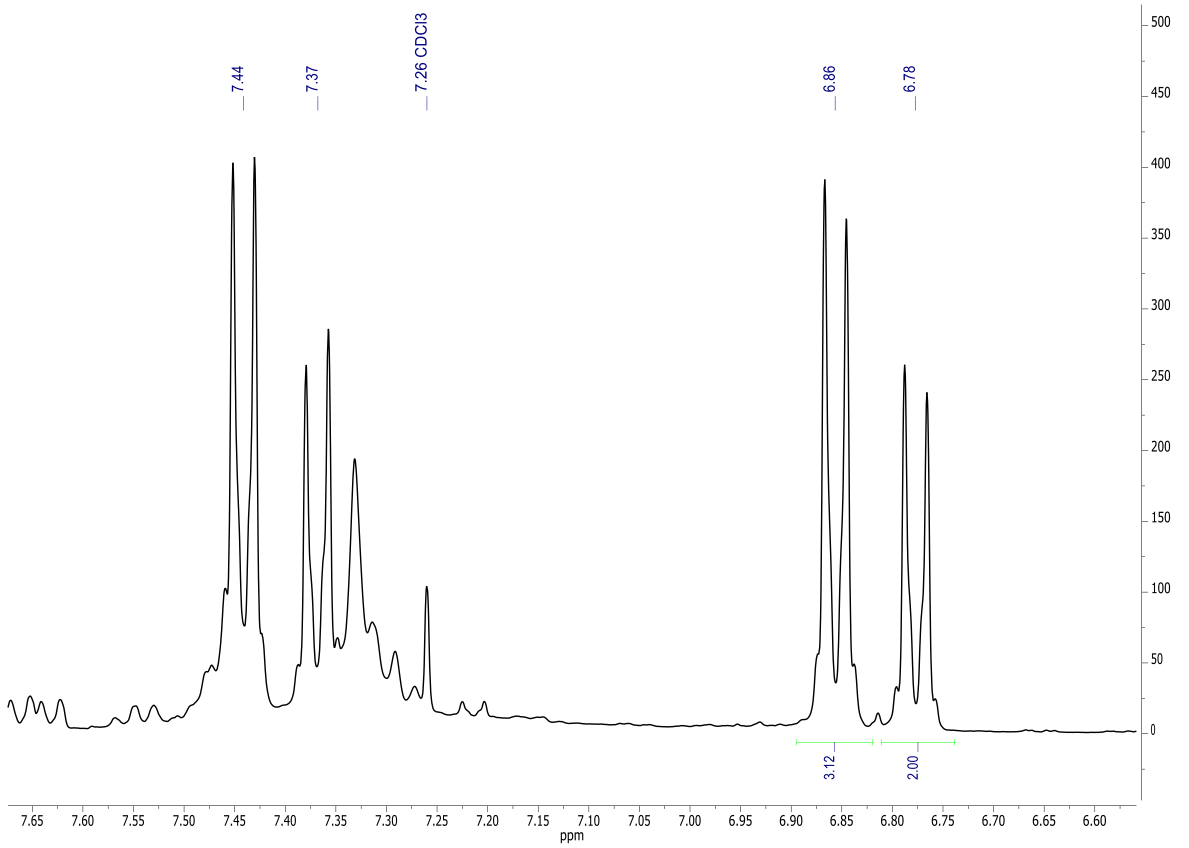

Figure S29. ^{1}H-NMR (400 MHz, 298 K, $CDCl_3$) of reaction 14.

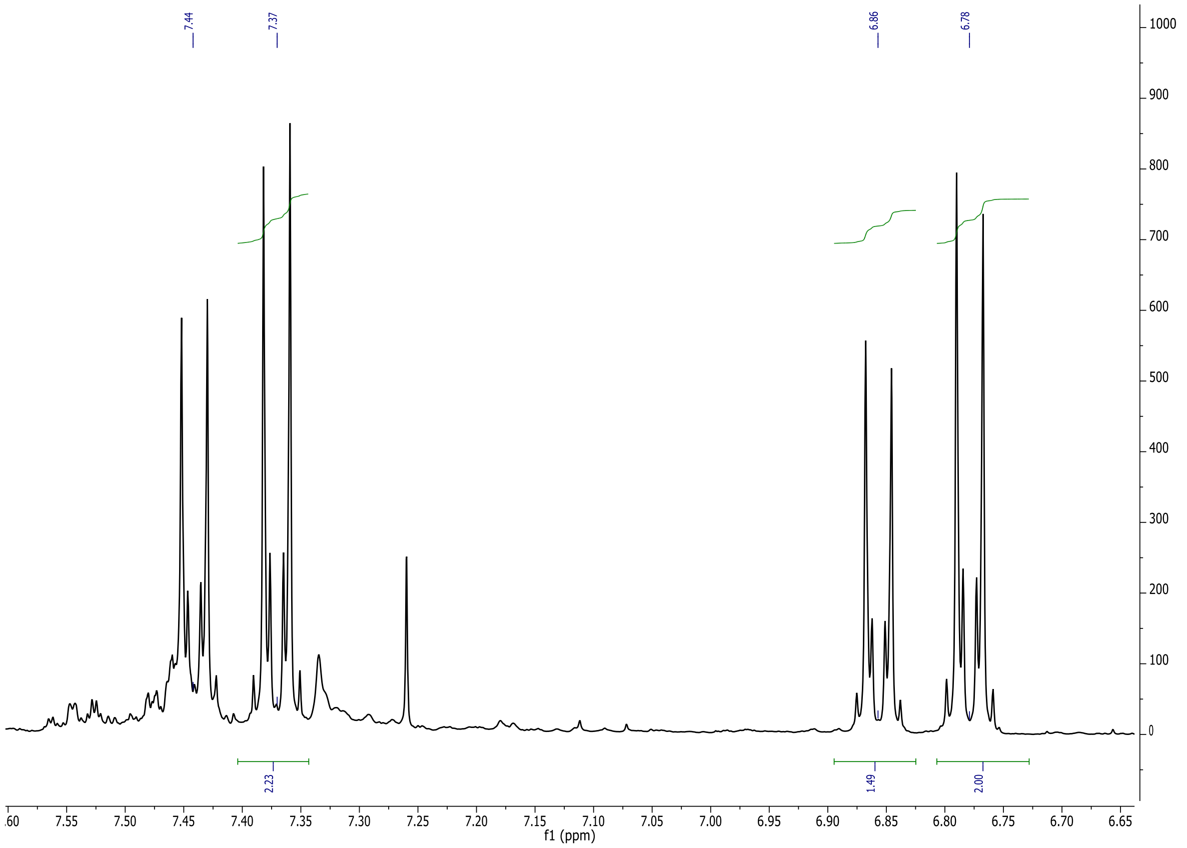

Figure S30 ^{1}H-NMR (400 MHz, 298 K, $CDCl_3$) of reaction 15.

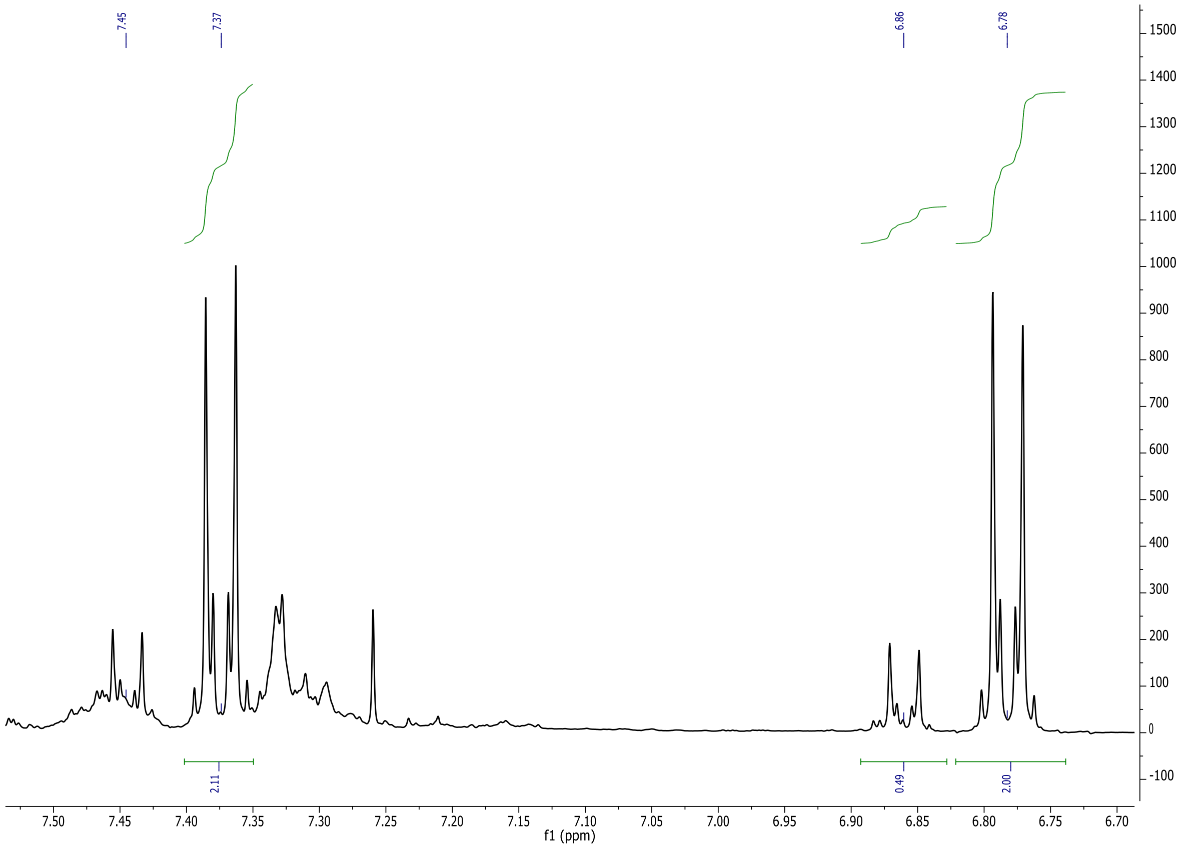

Figure S31. ^{1}H-NMR (400 MHz, 298 K, $CDCl_3$) of reaction 16.

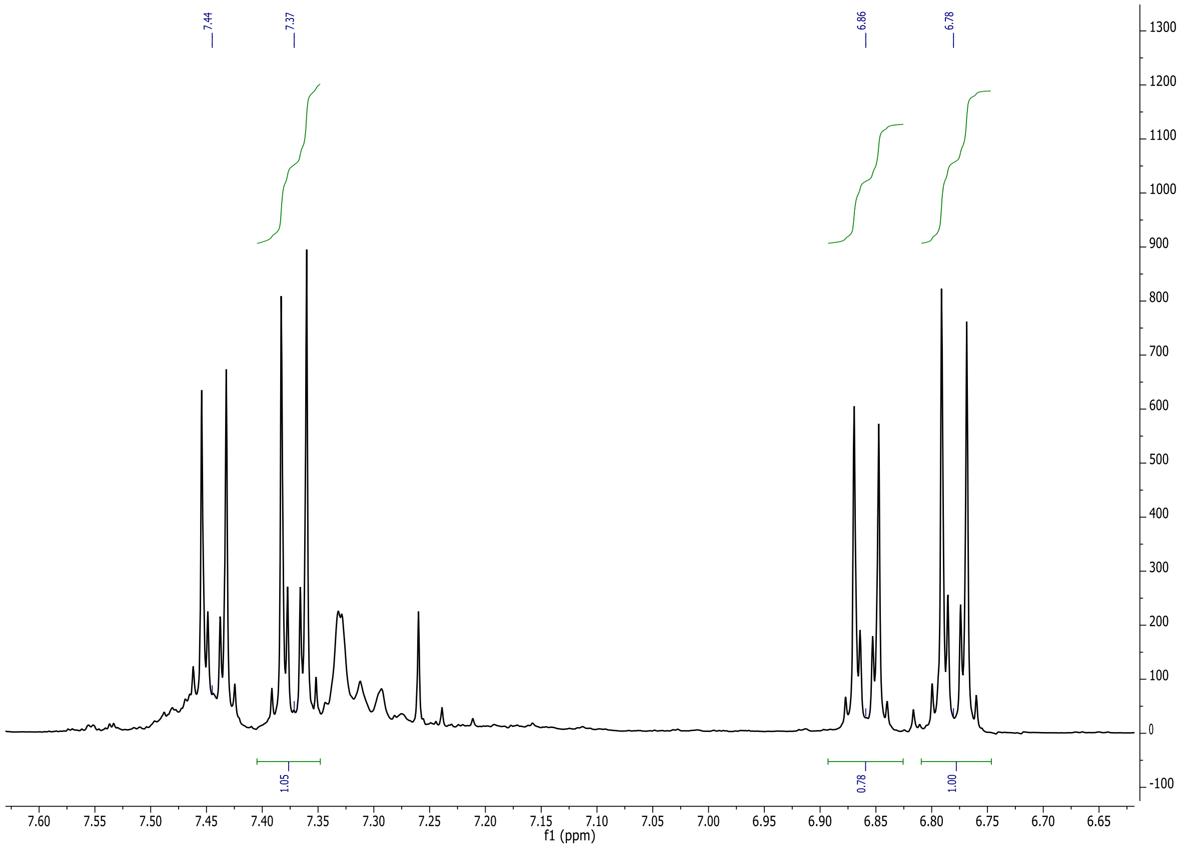

Figure S32. ^{1}H-NMR (400 MHz, 298 K, $CDCl_3$) of reaction 17.

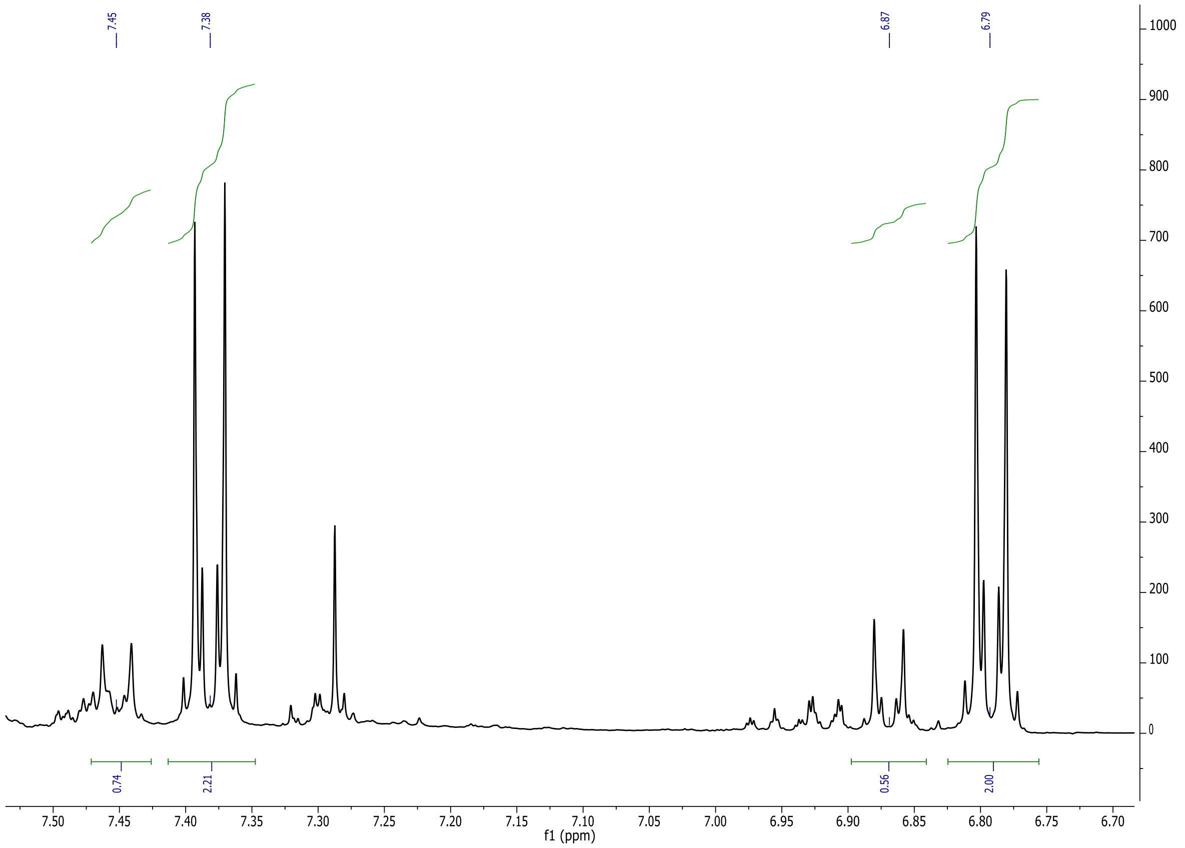

Figure S33. ^{1}H-NMR (400 MHz, 298 K, $CDCl_3$) of reaction 18.

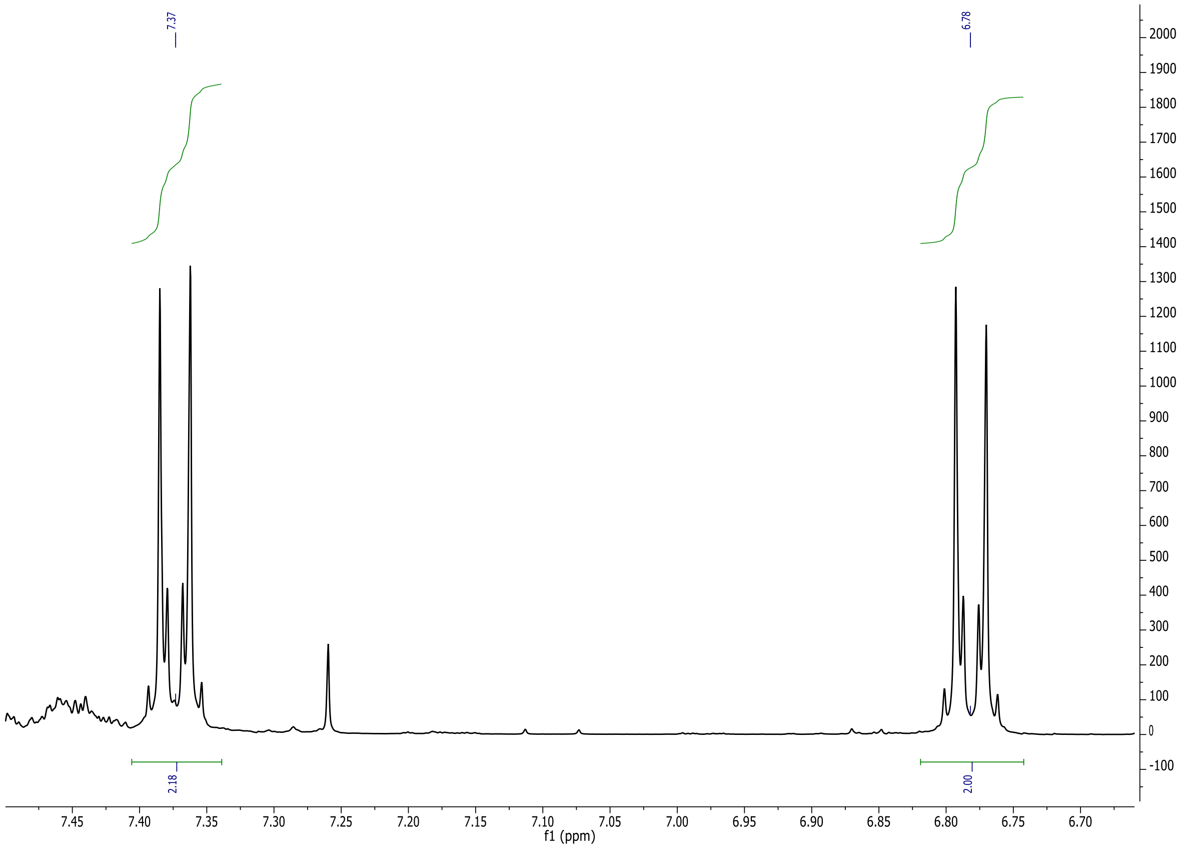

Figure S34. ^{1}H-NMR (400 MHz, 298 K, $CDCl_3$) of reaction 19.

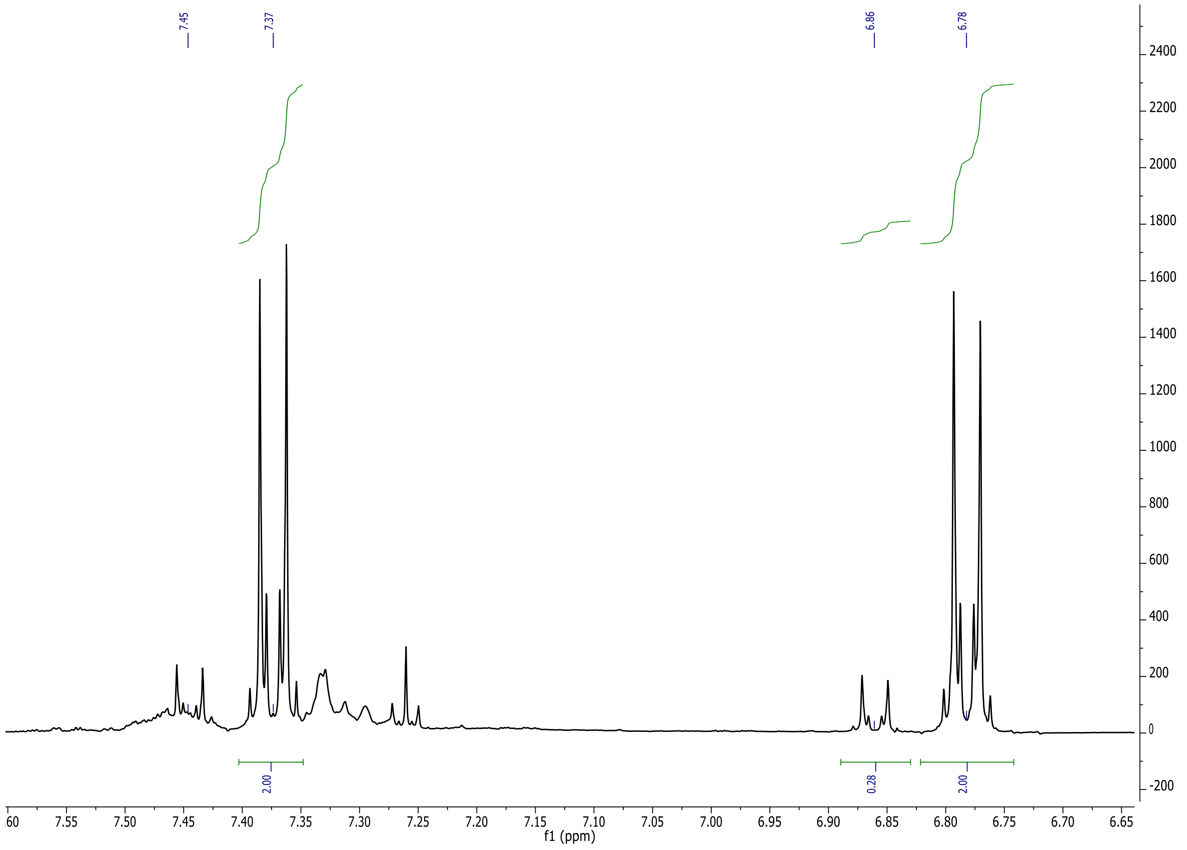

Figure S35. ^{1}H-NMR (400 MHz, 298 K, $CDCl_3$) of reaction 20.

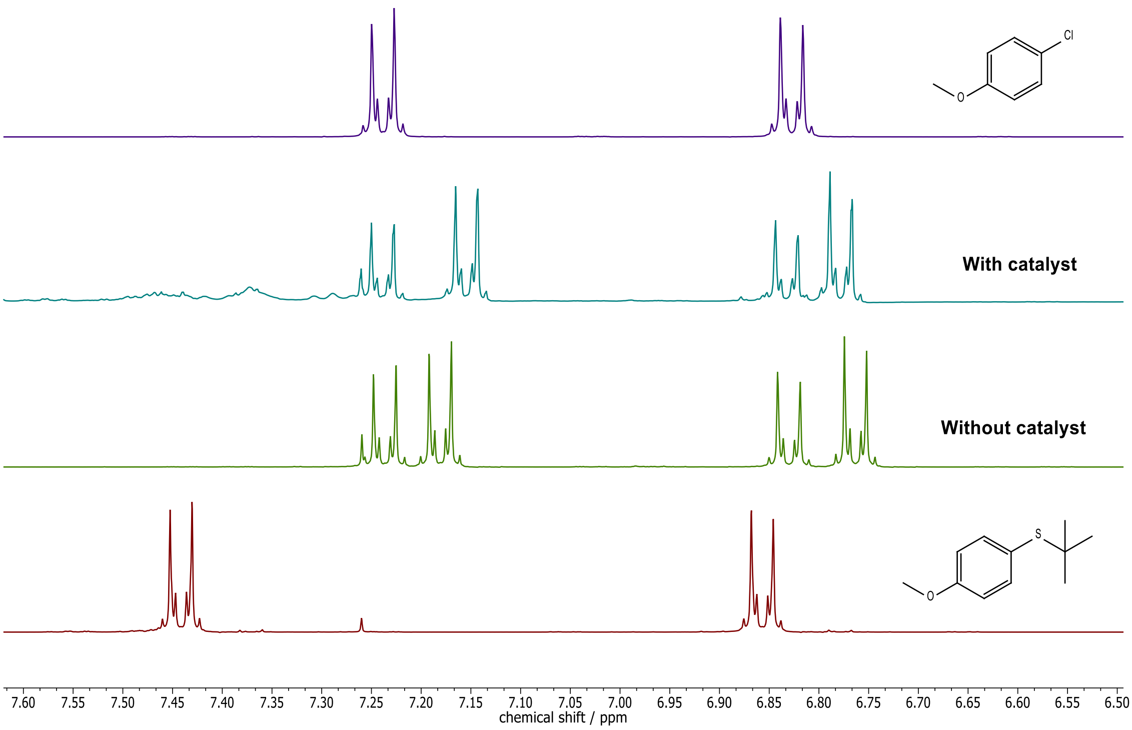

Figure S36. ^{1}H-NMR (400 MHz, 298 K, $CDCl_3$) of the reactions with 4-chloroanisole.

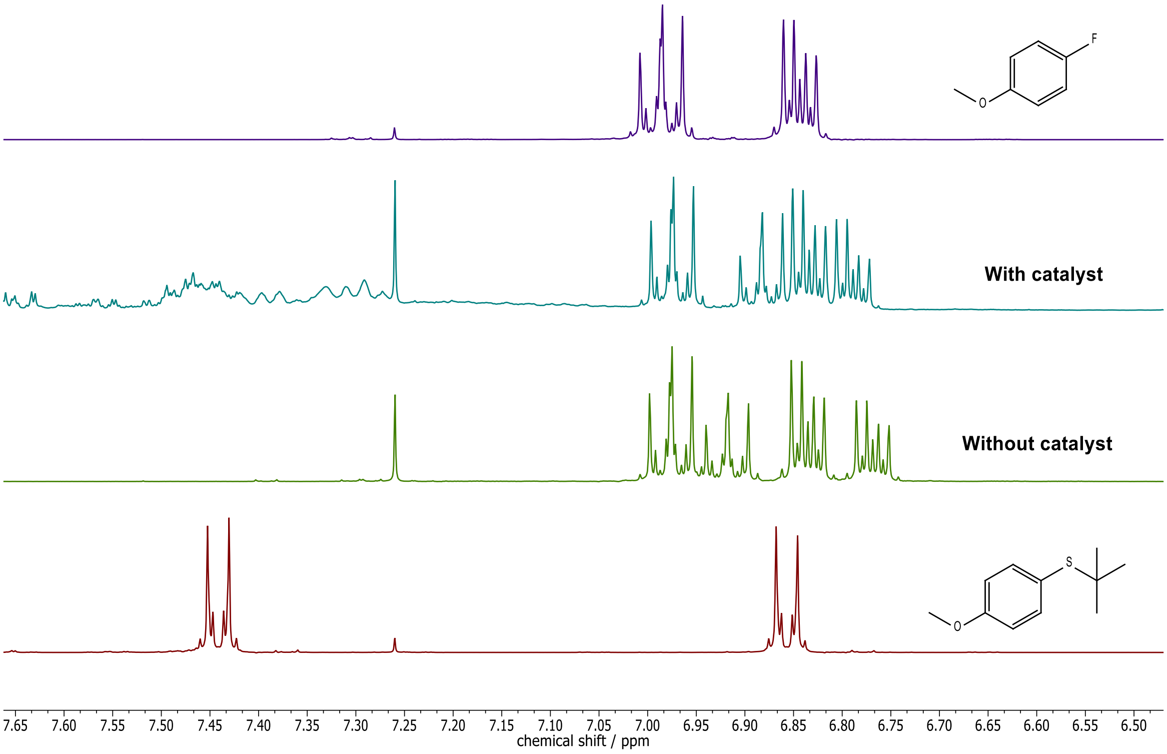

Figure S37. ^{1}H-NMR (400 MHz, 298 K, $CDCl_3$) of the reactions with 4-fluoroanisole.

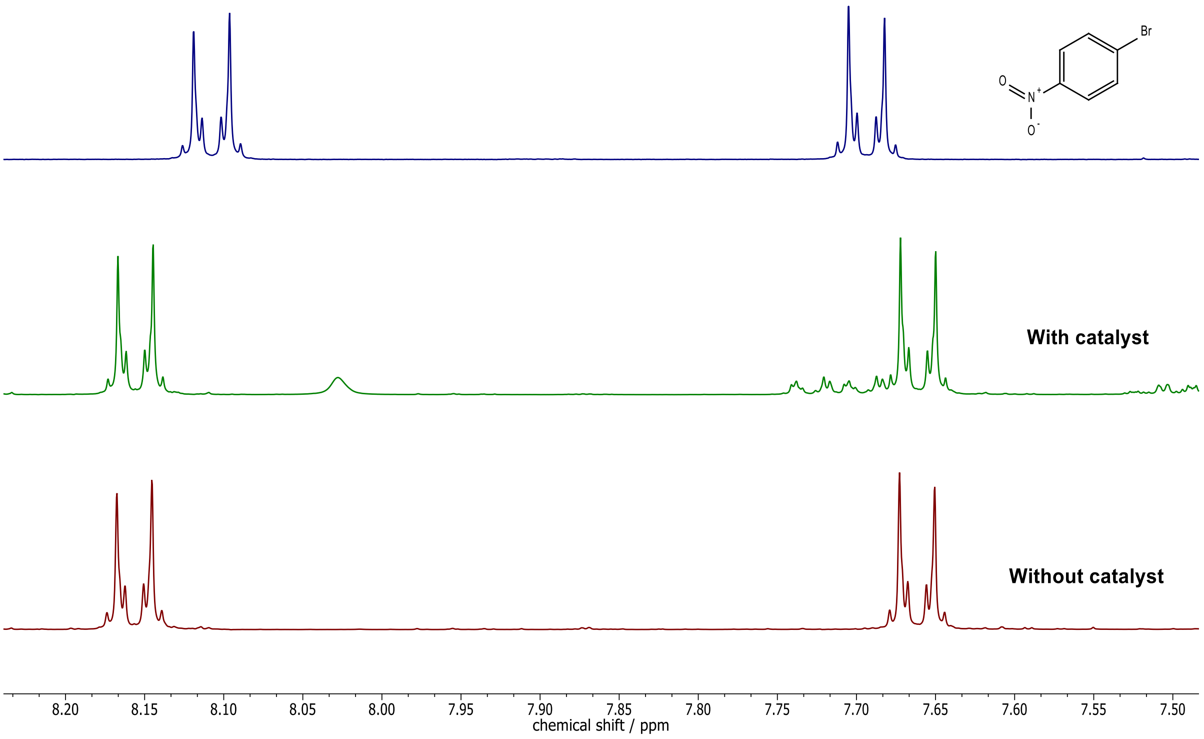

Figure S38. ^{1}H-NMR (400 MHz, 298 K, $CDCl_3$) of the reactions with 4-nitrobromobenzene

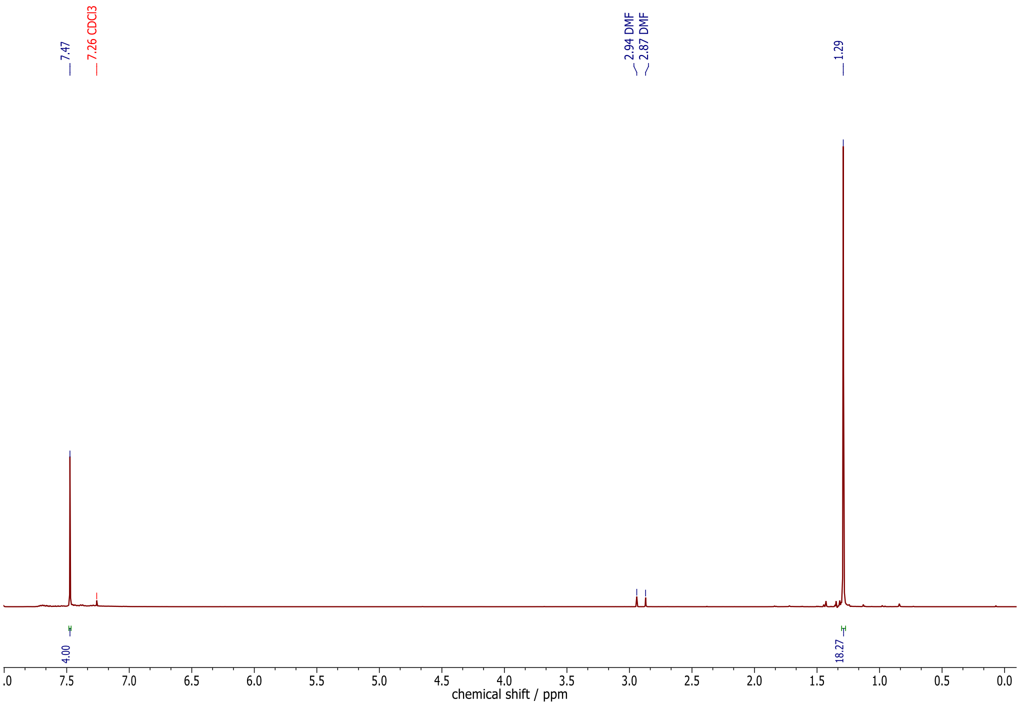

Figure S39. ^{1}H-NMR (400 MHz, 298 K, $CDCl_3$) of the reaction with 1,4-diiodobenzene at 50 °C.

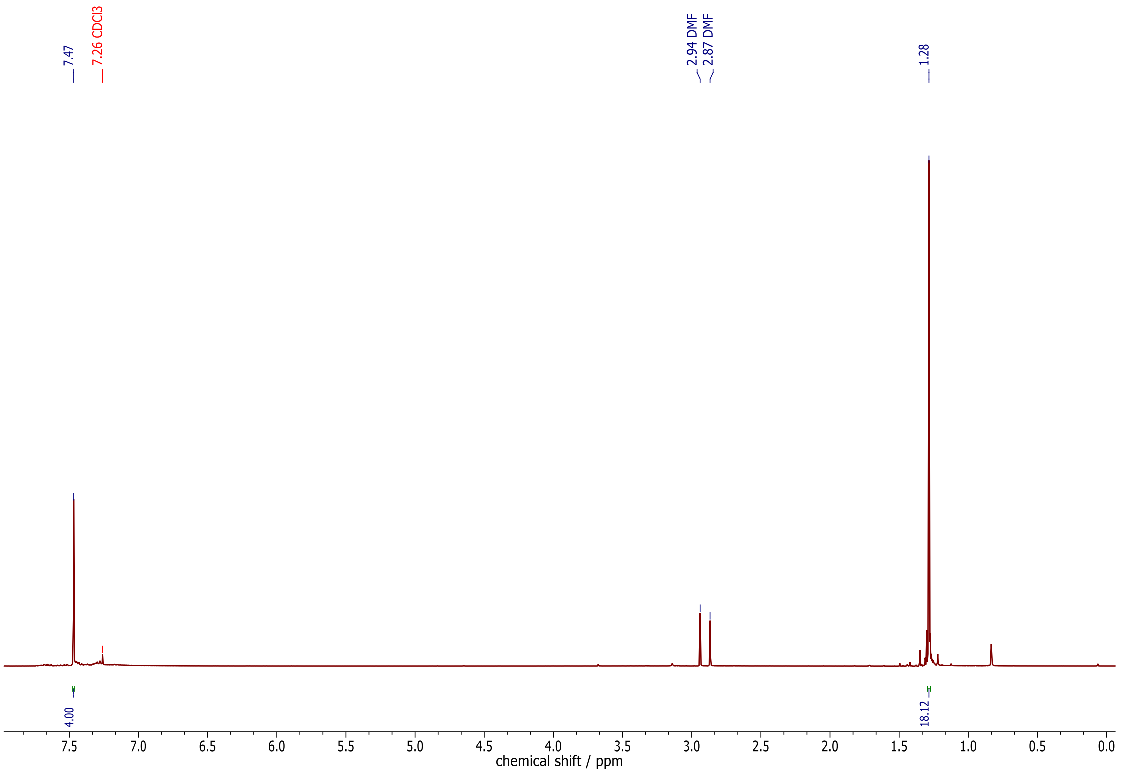

Figure S40. ^{1}H-NMR (400 MHz, 298 K, $CDCl_3$) of the reaction with 1,4-diiodobenzene at 80 °C.

3 Literature

(1) Fulmer, G. R.; Miller, A. J. M.; Sherden, N. H.; Gottlieb, H. E.; Nudelman, A.; Stoltz, B. M.; Bercaw, J. E.; Goldberg, K. I.; Gan, R.; Apiezon, H. NMR Chemical Shifts of Trace Impurities : Common Laboratory Solvents , Organics , and Gases in Deuterated Solvents Relevant to the Organometallic Chemist. *Organometallics* **2010**, *29*, 2176–2179.

(2) Wang, L.; Zhou, W.-Y.; Chen, S.-C.; He, M.-Y.; Chen, Q. A Highly Efficient Palladium-Catalyzed One-Pot Synthesis of Unsymmetrical Aryl Alkyl Thioethers under Mild Conditions in Water. *Adv. Synth. Catal.* **2012**, *354*, 839–845.

(3) Reddy, T. J.; Iwama, T.; Halpern, H. J.; Rawal, V. H. General Synthesis of Persistent Trityl Radicals for EPR Imaging of Biological Systems. *J. Org. Chem.* **2002**, 4635–4639.

(4) Cogolli, P.; Testafari, L.; Tingoli, M.; Tiecco, M.; *J. Org. Chem*. **1979**, *44*, 2636 – 2642.

(5) Bovonsombat, P.; Ali, Rameez; K., Chiraphorn; L., Juthamard; P., Kawin; Aphimanchindakul, S.; Pungcharoenpong, N.; Timsuea, N.; Arunrat, A.; Punpongjareorn, N.; *Tetrahedron* **2010**, *66*, 6928 – 6935.

(6) Furuya, T.; Kaiser, H.; Ritter, T.; *Angew. Chem. Int. Ed.* **2008**, *47*, 5993 – 5996.

(7) Wang, L.; Zhou, W.-Y.; Chen, S.-C.; He, M.-Y.; Chen, Q. A Highly Efficient Palladium-Catalyzed One-Pot Synthesis of Unsymmetrical Aryl Alkyl Thioethers under Mild Conditions in Water. *Adv. Synth. Catal.* **2012**, *354*, 839–845.
